# The Association between Food Security Status and the Home Food Environment among a Sample of Rural South Carolina Residents

**DOI:** 10.3390/nu15183918

**Published:** 2023-09-09

**Authors:** Cassius Hossfeld, Lior Rennert, Samuel L. K. Baxter, Sarah F. Griffin, Michelle Parisi

**Affiliations:** 1 Department of Public Health Sciences, Clemson University, Clemson, SC 29634, USA; liorr@clemson.edu (L.R.); baxter@clemson.edu (S.L.K.B.); sgriffi@clemson.edu (S.F.G.); 2 Cooperative Extension Service, Clemson University, Clemson, SC 29634, USA; michelle.parisi@uga.edu

**Keywords:** food security, home food environment, food pantry, rural

## Abstract

Prior research suggests that food security status may have an effect on the home food environment. Further, the literature suggests that food access factors may function to influence said relationship. The purpose of this research is to fill a gap in the literature on this relationship, as well as to identify potential food access effect modifiers. This research employs linear mixed effects modeling with a random intercept variable (zip codes). Eleven food access variables are included in regression analyses and are tested as potential effect modifiers in the association between food security status and the home food environment. Food security status is significantly associated with the home food environment (95% CI = 0.1–1.38) in the unadjusted model. In the adjusted model, food pantry usage is found to be a significant effect modifier on the association between food security status and the home food environment. This research concludes that food security status has a significant but disparate effect on the home food environment depending on participant food pantry usage. Practical implications from this research would be for relevant stakeholders to potentially improve rural food pantry access in order to increase the home food environment among rural and food insecure populations.

## 1. Introduction

Food security is the ability of one or more people to access the foods needed to live an active and healthy life [1]. In the U.S., food security status is typically measured through four separate categories: (1) high food security, (2) marginal food security, (3) low food security, and (4) very low food security [2]. This discrete measure allows researchers to separate individual/household food security status through the use of questionnaires and survey protocols, such as the six-item short form Household Food Security Survey (HFSS) [3].

The home food environment is a concept that has been defined by the availability of healthy/unhealthy foods in the home and the accessibility of healthy/unhealthy foods in the home [4]. This construct can be useful for identifying what food items a household consistently has in their home, indicating what household members will be eating on a daily basis [5]. The home food environment is typically measured by the amount of healthy/unhealthy food types accessible/available in a household over a recent time period [4,6]. This type of measure is useful because it relates the home food environment to concepts such as the demographic attributes associated with the social determinants of health, food security status, and food access. For example, it is found in the literature that factors such as gender [7], income [8], and obesity [9] are significant predictors of the home food environment. It is important to understand more about the home food environment and, specifically, how it associates and interacts with factors such as food security status, demographic attributes, and food access.

Considering the closely related nature of these two concepts, it should follow that those households with limited access to the foods needed to live an active and healthy life are the same households that have little accessibility to/available healthy food items in their homes. This research studies whether an association truly exists between these two concepts and, if so, what types of factors may influence such an association.

### 1.1. A Review of the Relationship between Food Security Status and the Home Food Environment

Connections between food security status and the home food environment can be found in the literature. For example, the literature supports the fact that employment status [10] and income [11] are predictors of food security status, while income [8] is also a predictor of the home food environment. Adams et al. [12] found a relationship between the home food environment and food security status among households with children during the COVID-19 pandemic. While their research identifies the changes in the home food environment based on changes in household food security status, the authors do not identify the association between household food security status and the home food environment [12]. Garasky et al. [13] do identify similar factors and find that the local (rural) food environment predicts food security status [13]. However, Garasky et al. [13] do not incorporate the home food environment in their research.

Nackers and Appelhans [14] recruited 41 parents to identify the association between food security status and the home food environment among households with children. Nackers and Appelhans [14] found that an obesogenic home food environment is significantly associated with reduced food security status. Adams et al. [6] identify the association between food security status and the home food environment of children (ages 5–18) during the COVID-19 pandemic. In their nationally representative study, the authors found that the total amounts of unhealthy food items in the home changed during the COVID-19 pandemic based on participant food security status; however, these changes were less pronounced for healthy food items [6]. Additionally, Shim, Hwang, and Kim [15] used the concept of a home food environment from Glanz et al. [16] to identify the effect of food security status on household food availability among an international rural sample outside the U.S. (Korea). Shim, Hwang, and Kim [15] found that a primary barrier to rural residents’ ability to improve their home food environment is living far away from food stores. While the authors in this study did not review the exact home food environment measures that were reviewed by Green and Glanz [4], Shim, Hwang, and Kim [15] uniquely identified the association between food security status and the home food environment among a rural sample. In sum, the aforementioned findings support the notion that food security status may have an effect on the home food environment.

While the prior studies suggest that there may be a general association between food security status and the home food environment, some of the most important factors discussed in the literature that influence this association are food access factors. For example, Garasky et al. [13] found that food access factors such as informal social support, proximity to a food site, and transportation are significant predictors of food security status. Further, Nackers and Appelhans [14] suggest that participants in their study were likely relying on food assistance to offset food insecurity by buying fruits and vegetables. Similarly, Shim, Hwang, and Kim [15] suggest that those households using public assistance and those households living farther away from food sites had significantly different food security statuses than those households with the opposite condition. The literature suggests that food access factors such as proximity to a food site, social support from family and/or friends, and public assistance may exert significant influence on the association between food security status and the home food environment. Therefore, in this research, it will be important to not only measure any potential associations between food security status and the home food environment among a rural sample but also to analyze the effects that separate food access factors may have on said relationship.

### 1.2. Food Access and the Social–Ecological Model

Charreire et al. [17] note that the social–ecological approach is beneficial when studying the food environment, as this framework can address varying individual-level and environmental-level influences. Andress [18] does exactly this by incorporating the social–ecological model to identify the ways by which food access influences food security and dietary practices among a rural population. In their research, Andress [18] notes that in the context of the influence that food access has on food security, the social–ecological model can be reduced to five food access dimensions: acceptability, accessibility, accommodation, affordability, and availability. These dimensions exist at both the individual level (affordability and acceptability) and at the environmental level (availability, accessibility, and accommodation). Caspi et al. [19] suggest that these five dimensions not only relate to the effect that food access has on food security but that these five dimensions are also a conceptualization of the food environment. Therefore, a brief review of the literature encourages the use of food access factors in any model that seeks to identify the effect of food security on the home food environment.

### 1.3. Research Questions/Hypotheses

This research seeks to address the following question:

(1) Is food security status associated with the home food environment of South Carolina residents from three rural counties? Further, in order to determine the differential effect that food security status may have on the home food environment based on varying levels of each food access factor, this research includes two additional research questions with stratified subsamples;
(2) Is food security status associated with the home food environment of food-secure South Carolina residents from three rural counties?
(3) Is food security status associated with the home food environment of food-insecure South Carolina residents from three rural counties?

Based on the prior literature [6,12,14,15], the research hypothesis for each of the three research questions is that a higher food security status of residents from three rural SC counties will associate with healthier home food environments when using linear mixed-effects modeling with zip codes as the random intercept variable.

## 2. Materials and Methods

### 2.1. Sample

This research is a part of the Clemson University portion of the High Obesity Program (HOP) Centers for Disease Control (CDC) funded grant that seeks to address healthy food access and physical activity in U.S. counties with an adult population that is at least 40% obese [20]. Hampton County, Lee County, and Marion County, SC, are three counties that have been identified by the HOP as meeting the above criteria [21]. Although not geographically connected, these three counties share the status of being rural [22], consistently low-income [23], and experiencing relatively low food security [24].

In total, twenty-one separate predictors (nine demographic variables, one health measure, and eleven food access variables) have been selected for inclusion in the final model and to test for effect modification (with food security status as the interacting term). The selection of the eleven food access variables can be theoretically justified through the social–ecological model [18]. Through the second and third research questions, the data are split into two distinct samples from the broader 2022 survey population: a sample of respondents who indicated food security (N = 211) and a sample of respondents who indicated food insecurity (N = 174). These separate samples were distinguished from the broader population in an attempt to separately identify the effect of food security status on the home food environment among food-secure and food-insecure households, using linear mixed-effects modeling with zip codes as the random intercept variable.

Through convenience sampling, the HOP research team at Clemson University administered 436 surveys throughout three rural SC counties. Specifically, in order to identify the needs in these three counties pertaining to obesity prevention, these surveys examine the topics of demographic information, food access, food security, the home food environment, physical activity, neighborhood safety, stress, and community belonging. Surveys were administered in person throughout the three counties by Clemson University research team members. Clemson University research team members would recruit participants on-site and administer paper copy surveys or allow participants to self-administer. Participants were compensated with a store credit gift card upon survey completion. These data were collected through convenience sampling procedures, and response rates were not obtained.

Cross-sectional data were collected in 2022 by a Clemson University research team partnering with the Centers for Disease Control (CDC) High Obesity Program (HOP). For the entire survey population (N = 436), the distribution of adult survey respondents by county is Hampton County, SC (N = 155); Lee County, SC (N = 129); and Marion County, SC (N = 151). For the survey sample subset including only those respondents indicating food security (N = 211), the distribution of adult survey respondents by county is Hampton County, SC (N = 84); Lee County, SC (N = 67); and Marion County, SC (N = 59). For the survey sample subset including only those respondents indicating food insecurity (N = 174), the distribution of adult survey respondents by county is Hampton County, SC (N = 55); Lee County, SC (N = 47); and Marion County, SC (N = 72).

### 2.2. Inclusion and Exclusion Criteria

The inclusion criterion for these survey data included those residents, at least eighteen years of age, living (at the time of the survey) in Hampton, Lee, or Marion County, South Carolina. The exclusion criterion for these survey data was simply the inverse of the inclusion criterion. Participant eligibility was checked by asking each participant to provide their zip code and age at the beginning of the survey. The following questions were asked to each participant at the beginning of each survey in order to ensure they lived in the appropriate counties under study: (1) What county do you live in? (2) What is your zip code?

### 2.3. Random Intercept and Predictor Variables

The random intercepts in the linear mixed-effects models in this research are zip codes. In the United States, ZIP (zone improvement plan) codes are five-digit numerical sequences that are specifically designated to a geographical area and are most frequently used for postal delivery [25]. This is why zip codes are useful as a random intercept variable in linear mixed-effects modeling, as the populations living within each zip code are relatively randomly distributed. Through the 2022 HOP survey protocol, respondents were asked to provide the zip code within which they live at the time of survey completion. In total, there were sixteen separate zip codes provided by respondents between the three rural SC counties.

This research included nine demographic predictor variables for analysis and one health measure. These demographic predictor variables included age, number of children in the household, educational status, employment status, ethnicity, gender, income, marital status, and race. One health measure, body mass index (BMI), was also included as a predictor variable for analysis. These variables were selected because they have been found in the literature to be associated with food security status [10,11] and the home food environment [7,9].

This research comprised eleven food access predictor variables for analysis. These food access predictor variables included food pantry usage, free meal usage, school meal usage, reliance on hunting/fishing for food, reliance on friends/coworkers/neighbors for food, reliance on relatives outside the home for food, food stamp usage, store access, number of SNAP vendors in the zip code, number of healthy SNAP vendors in the zip code, and number of unhealthy SNAP vendors in the zip code. Food pantries are food distribution centers where households can collect free/reduced-price food [26]. Typically, the households accessing food pantries are low-income. SNAP (Supplemental Nutrition Assistance Program) is a federal program that supports low-income households with nutrition benefits in the form of “stamps” (similar to pre-paid debit cards) that can be used at designated food sites for healthy food purchases [27]. The eleven food access predictor variables were selected for their status as food access variables that were similarly found in the prior literature [18].

### 2.4. Outcome Variable

The outcome variable in this research is the home food environment. This is measured as a continuous (interval) variable ranging from 0 to 18, whereby the zero point is meaningless (the zero point does not indicate the absence of food in the home). The home food environment is a concept that has been previously validated in the literature [4]. This variable is based on four measures: availability of healthy foods in the home, availability of unhealthy foods in the home, accessibility of healthy foods in the home, and accessibility of unhealthy foods in the home. For the availability of healthy foods in the home, participants responded (yes/no) to survey questions asking about the availability of healthy food items (i.e., bananas, apples, grapes, carrots, tomatoes, dark leafy greens, reduced-fat hotdogs, whole-grain bread, low-fat milk, diet soda) in their home in the past week. For availability of unhealthy foods in the home, participants responded (yes/no) to survey questions asking about the availability of unhealthy food items (i.e., candy/cookies, snack chips, regular whole milk, regular soda, regular hot dogs, white bread) in their home during the past week. Accessibility of healthy and unhealthy foods in the home was defined by participant responses (never or rarely, sometimes, often, almost always) to survey questions asking about how often healthy food items such as fruits and vegetables are in the refrigerator or available on the counter, and how often unhealthy food items such as candy or chips and ice cream/cake/pastries/ready-to-eat sweet baked goods are available to eat in the home. These variables were recoded and summed to create the home food environment variable. This summary measure is a continuous variable with values ranging from 0 to 18 where higher values indicate a healthier home food environment. The values are a means of comparison; therefore, each value only has meaning when compared with another value from the same score. Thus, while the intervals between each value are meaningful, the value itself (such as zero) is not. For example, a score of less than 8.5 indicates that the household had more unhealthy food items available/accessible than healthy food items, while a score of greater than 8.5 indicates the household had more healthy food items available/accessible than unhealthy food items. A score of exactly 8.5 indicates that the household had an even amount of healthy and unhealthy food items available/accessible in the home. The values associated with the outcome variable essentially represent how many healthy/unhealthy food items are typically accessible in the participant’s home (the question did not specify a time period), as well as how many healthy/unhealthy food items were available in the participant’s home within a week prior to survey completion. Therefore, the summary measure is a proxy measure for the home food environment, that is, how many separate healthy/unhealthy food types are accessible/available in the participant’s home at a given time. Past research has operationalized food availability (seven items) and food accessibility (twelve items) separately to define the home food environment [4]. This study followed the coding practices of Green and Glanz [4] to develop a composite measure of the home food environment incorporating both food accessibility and food availability.

### 2.5. Explanatory Variable

The primary explanatory variable of interest in this research is food security status. This is measured as a categorical (dichotomous) variable ranging from 0 to 1. Food security status is defined as participant responses to six separate United States Department of Agriculture (USDA) questions on the participant’s ability to adequately afford/obtain enough healthy food. These measures are subjective, as they ask the participant to use their judgment on what “healthy” and “enough” food is for their household. The development of the food security status explanatory variable in this research was guided by the validated USDA six-item short form HFSS [3]. For the purposes of this research, the food security status explanatory variable was recoded into a binary variable indicating (0) food insecurity and (1) food security. This was done in response to the relatively small sample size, as the four-category measure (high food secure, marginal food secure, low food secure, and very low food secure) provided too few observations per category. The dichotomization of the four-category food security variable into a two-category variable is supported by the USDA HFSS [3].

### 2.6. Demographic Variables and Health Measure

Ethnicity, gender, marital status, and race were recoded into binary variables. It is important to note that there were only four participants who classified their race as “other” (neither Black nor White). Therefore, these four race responses were omitted. Ultimately, the race variable used in regression analysis comprises those respondents identifying as Black (N = 288) or White (N = 135). This research calculated participant body mass index (BMI) using data collected on participant height and weight. BMI values were calculated using the CDC formula [28]. Education status was coded with the following categories: no high school degree, high school degree through associate degree, bachelor’s degree, and master’s degree or higher. Income was coded with the following categories: less than USD 10,000 annually; between USD 10,000 and USD 19,999 annually; between USD 20,000 and USD 34,999 annually; between USD 35,000 and USD 49,999 annually; between USD 50,000 and USD 74,999 annually; and USD 75,000 or more annually. Employment status was coded with the following categories: employed/self-employed; out of work (out of work for >1 year + out of work for <1 year); retired; unable to work; and other. The number of children in the household was coded in numerical order: zero children, one child, two children, and three or more children.

### 2.7. Food Access Variables

In their research, Andress [18] used the social–ecological model to discern between five separate dimensions of food access: availability (sources of food), accessibility (using available food), affordability (ability to pay for food), accommodation (food sources’ ability to fit consumer needs), and acceptability (consumer attitudes toward food sources). Using the social–ecological model adapted from Andress [18], eleven separate food access predictor variables selected from the High Obesity Prevention survey protocol fit with four of the aforementioned five food access dimensions. Food pantry usage, free meal usage, school meal usage, reliance on hunting/fishing for food, reliance on friends/coworkers/neighbors for food, and reliance on relatives outside the home for food are all characteristics of the availability food access dimension. This is because the availability of food access dimension is “defined as the adequacy of the supply of healthy food” [18]. Store access is a characteristic of the accessibility food access dimension. This is because the accessibility food access dimension is defined as “the geographic location of the food supply and ease of getting to that location” [18]. The number of total/healthy/unhealthy SNAP (Supplemental Nutrition Assistance Program) vendors is characteristic of the accommodation food access dimension. This is because the accommodation food access dimension is defined as “representing how well local food sources accept and adapt to local residents’ needs” [18]. Food stamp usage is a characteristic of the affordability food access dimension. This is because the affordability food access dimension is defined as “representing food prices and people’s perceptions of worth relative to food cost” [18]. The acceptability food access dimension is not used for any predictor variables in this research. This is because the dimension “is defined as an individual’s attitude regarding the attributes of their local food environment and whether or not the given supply of products meets their personal standards” [18]. The social–ecological model, as incorporated by Andress [18], helps to justify the use of eleven food access predictor variables used in this research by categorizing food access factors by five separate food access dimensions. The incorporation of the social–ecological model in this research uses previously peer-reviewed literature [18] to substantively justify why each food access predictor can function as a factor in the pathway relating food security status with the home food environment.

Food pantry usage, free meal usage, school lunch usage, reliance on hunting/fishing for food, reliance on friends/coworkers/neighbors for food, and reliance on relatives outside the home for food are survey items derived from the validated 88-item “Mapping the Food Environment” survey [29]. These items asked participants to indicate how often they had utilized various food resources within the past three months (of taking the survey). These were a series of five-category Likert scale survey questions. These survey questions were all recoded into binary variables so that the five-category Likert scales were reduced to two categories. The original five-category variables included the following responses to answer the question of whether a participant’s household had depended on a particular food source in the past three months: never; less than once a month; once a month; 2–3 times a month; every week. These responses were recoded into a binary variable with the following combined categories: never + less than once a month; once a month + 2–3 times a month + every week. This recoding procedure was initially selected for the food pantry usage item specifically because the food items received from a food pantry will typically provide a household with balanced meals for three days [30]. This coding procedure was then applied to similar items, asking participants about their use of food resources (food pantry usage, free meal usage, school lunch usage, reliance on hunting/fishing for food, reliance on friends/coworkers/neighbors for food, and reliance on relatives outside the home for food) for the purposes of consistency.

Store access was coded in this research as a combination of the following items from the 2022 HOP survey protocol: the form of transportation the respondent uses to arrive at the food site they most often use for food (1 = car, 2 = other form of transportation); how long it would take (in minutes) for the respondent to arrive at the food site they most often use for food if they were to walk (ranging from 1 = “30 min or more” to 4 = “10 min or less”); how important it is for the respondent to shop for food at a food site near where they live (ranging from 1 = “very important” to 4 = “not at all important”); how important it is for the respondent to shop for food at a food site near where they spend most of their time (ranging from 1 = “very important” to 4 = “not at all important”). The coding procedure for this predictor variable was informed by the methods used by Green and Glanz [4].

The two separate continuous predictor variables representing the number of healthy/unhealthy SNAP vendors in each zip code were derived from definitions of SNAP store-type categories provided by the USDA Food and Nutrition Services (FNS) [31]. Further, the USDA FNS similarly provided a comprehensive list of SNAP vendors by zip code in all South Carolina counties [31]. For the purposes of this research, the definitions of healthy and unhealthy SNAP vendors were determined by the following criteria: food sites categorized as “large grocery store”, “medium grocery store”, “small grocery store”, “supermarket”, “super store/chain store” were defined as healthy; food sites categorized as “convenience store”, “combination grocery/other” were defined as unhealthy.

### 2.8. Statistical Analyses

Descriptive analyses for this research included identifying the mean, standard deviation, median, and range for continuous variables and identifying the N (%) for each categorical variable and the corresponding categories within.

Linear mixed effects models (LMM) with a random intercept for zip code (to account for within-site sampling correlation) were used to evaluate the relationship between food security and other key predictors of the home food environment. The reason for using LMM was because of the nature of the convenience sampling data collection methods employed. To adjust for potentially highly correlated observations, specifically those observations that were collected at the same site, zip codes were included as the random intercept in the analysis.

Interaction terms between each of the twenty-one separate predictor variables (nine demographic variables, one health measure, and eleven food access variables) were generated with the explanatory variable in order to check for effect modification. If the interaction term is statistically significant in a model with the two independent variables comprising the interaction term, then the interaction term is concluded to be an effect modifier in the relationship between food security status and the home food environment. LMM were also used to test for effect modification.

Three separate datasets were developed from this study: one with the entire sample, one with only the respondents who indicated food security, and one with only the respondents who indicated food insecurity. The reason for doing this was to test for differences between the stratified groups, as demographic variable outcomes and/or food access variable outcomes may differ by respondent’s food security status. In total, six separate types of models were run in the regression analysis: fully unadjusted models for the entire sample; fully unadjusted models for the subsample of respondents who indicated food security (Table S1); fully unadjusted models for the subsample of respondents who indicated food insecurity (Table S2); a fully adjusted model for the entire sample; a fully adjusted model for the subsample of respondents who indicated food security (Table S3), and a fully adjusted model for the subsample of respondents who indicated food insecurity (Table S4). For the context of this article, fully unadjusted models only have one predictor variable in each model, while fully adjusted models have all of the predictor variables and significant interaction terms in each model.

Regression analysis controlled for demographic/food access factors and was conducted using R software version 4.3.1 [32]. The following R packages were used for analysis: haven; cat; car; table1; tidyverse; tibble; gapminder; highcharter; knitr; kableExtra; dplyr; epiDisplay; expss; nlme; tidyr; dplyr; ggplot2; readx1; emmeans; sjstats; lme4; lmerTest; MuMIn; xtable; broom; broom.mixed; readx1; janitor; irr; and performance [32].

## 3. Results

### 3.1. Descriptive Statistics

From the total sample, 436 observations were analyzed in this study (Table 1), including 66% Black respondents and 31% White respondents. A majority of respondents were food secure (48%), and the largest income group was those earning between USD 20,000 and USD 34,999 annually (23.6%). The mean body mass index among respondents was 31.3, which is higher than the U.S. adult population mean of 26.6 9 [33]. A majority of respondents neither used a food pantry service nor a free meal service within the past three months. Further, the mean home food environment score was 10.1.

Table 1 provides descriptive statistics and frequencies for the total sample, as well as for respective subsamples, including only those food-secure respondents and those food-insecure respondents. As can be seen in Table 1, among the subsample of respondents who indicated food security, a majority indicated high food security (72.5%) as compared with marginal food security (27.5%). Further, the largest income group earned more annually among the subsample of respondents who indicated food security as compared with that of the total sample, with the largest income group among those food secure respondents earning between USD 50,000 and USD 74,999 annually (18%). Additionally, the largest income group was the same between the total sample and the subsample of respondents who indicated food insecurity, with the largest income group among these respondents being those earning USD 20,000 to USD 34,999 annually (30.5%). Table 1 also shows that among the subsample of respondents who indicated food insecurity, a majority indicated low food security (56.9%) as compared with very low food security (43.1%). An interesting note on the subsample of respondents who indicated food insecurity is that a majority of these respondents suggested that they had used a food pantry within the prior three months of survey completion (62.1%). This is a higher proportion than those respondents from the total sample (44%) and the subsample of respondents who indicated food security (31.3%).

### 3.2. Effect Modification

Table 2 shows the twenty-one variables that were tested for effect modification in the relationship between food security status and the home food environment. Of these twenty-one potential effect modifiers, two were found to truly modify the relationship: food pantry usage and race.

### 3.3. Regression Results

Table 3 shows the fully unadjusted models for the entire sample. Noteworthy findings from this table include the fact that food insecurity has a significantly negative association with the unadjusted home food environment (95% CI = −1.38–0.01) among the entire sample. Further, items such as age, education status, food stamp usage, and income share significant associations with the unadjusted home food environment. For example, having a bachelor’s degree (95% CI = 0.85–2.9) or a master’s degree or higher (95% CI = 0.48–2.91) shares a significantly positive association with a higher unadjusted home food environment score as compared with those without a high school degree. Similarly, having used food stamps in the twelve months prior to survey completion (95% CI = −1.72–−0.41) shares a significantly negative association with the unadjusted home food environment as compared with not having used food stamps in the twelve months prior to survey completion. As seen in Table 3, income has a significant effect on the unadjusted home food environment, as earning at or more than USD 75,000 annually (95% CI = 1.24–3.57) shares a positive significant association with an increased unadjusted home food environment as compared with earning less than USD 10,000 annually. This finding supports those presented in the literature [8].

Table 4 shows the fully adjusted model for the entire sample. Table 4 also includes the interaction terms representing the interaction between food security status and race and the interaction between food security status and food pantry usage. An interesting finding from the fully adjusted model (representing the entire sample) is the significant associations between educational attainment and the adjusted home food environment. For example, those respondents with a bachelor’s degree (95% CI = 0.51–5.09) and a master’s degree or higher (95% CI = 0.70–5.97) reported significantly higher adjusted home food environment scores as compared with those respondents without a high school degree. It can be inferred from the data that post-secondary educational attainment is associated with an improved adjusted home food environment. An additional finding to note from the adjusted model is that the food security status and food pantry usage interaction term is statistically significant (95% CI = −4.96–−0.19). This provides further evidence for the fact that the association between food security status and the home food environment may differ between varying degrees of participant food pantry usage. When reviewing the unadjusted tables that are stratified by subsample (Tables S1 and S2), we find that among food secure respondents, not using a food pantry is positively associated with respondent home food environment scores (95% CI = −0.20–1.56). However, among food insecure respondents, not using a food pantry is negatively associated with respondent home food environment scores (95% CI = −2.59–0.55). These findings further support the notion that food pantry usage modifies the effect that food security status has on the home food environment.

## 4. Discussion

The home food environment refers to the types (healthy/unhealthy) of foods accessible and available in the home. This is an important indicator of whether or not household members are eating nutritious foods on a daily basis [5]. This study measured the home food environment in terms of the availability of healthy foods in the home, availability of unhealthy foods in the home, accessibility of healthy foods in the home, and accessibility of unhealthy foods in the home. This research finds that among a sample of rural South Carolina residents, there is a significant and positive association between food security status and the unadjusted home food environment (*p* = 0.02). This research also finds that a specific food access factor, food pantry usage, can modify the effect of food security status on the home food environment. This finding is significant because it suggests that the frequency with which a survey participant has used a food pantry within three months prior to taking the survey (less than once a month or at least once a month) significantly affects the relationship between food security status and the home food environment among the participant’s responses. It should be noted that prior research does not address this concept. The finding that food security status has a disparate effect on the home food environment based on varying levels of food pantry usage is a novel finding emanating from our study.

These results support prior research suggesting an association between food security status and the home food environment [6,14]. Adams et al. [6] measured the home food environment in terms of total food in the home: high-calorie snack foods, desserts and sweets, fresh foods, and nonperishable processed foods. Their findings suggested that food-secure households were less likely to decrease in four of the five categories during the COVID-19 pandemic as compared with food-insecure households [6]. The only category where food insecure households were more likely to increase the total amount during the COVID-19 pandemic was with nonperishable processed foods [6]. These findings differ from prior research in so far as this research sums all food types along a single range of a high quantity of unhealthy foods to a high quantity of healthy foods. However, this research shares similarities with prior findings by identifying overlapping significant associations between increased food security and increased amounts of healthy foods in the home and decreased food security and increased amounts of unhealthy foods in the home [6]. Nackers and Appelhans [14] measured the home food environment using an “obesogenic home food availability score,” which included the following food items: snacks, frozen foods, caloric beverages, desserts, candy, kitchen access, and refrigerator access. Their findings suggested that respondents with higher food security values had lower obesogenic scores [14]. Nackers and Appelhans’s [14] findings on obesogenic scores are similar to the findings from this study that greater obesogenic scores (a measure of unhealthy foods) are significantly associated with lower food security status scores. Interestingly, however, Nackers and Appelhans [14] also find that fruit and vegetable availability in the home is not significantly associated with much variation in respondent food security status. In their research, Nackers and Appelhans [14] consider whether these findings may be due to the ability of food-insecure households to access fruits and vegetables at a similar rate as food-secure households due to food assistance. The authors note that prior research has suggested similar findings where fruit and vegetable intake did not significantly vary by respondent food security status [34]. Ranjit et al. [35] suggest a similar finding by noting that home food environment measures tend to explain the consumption of unhealthy foods.

This research also supports prior literature suggesting an association between food security status and the home food environment among a rural sample found outside the United States [15]. In their research, Shim, Hwang, and Kim [15] measured the home food environment in Korea through the number of food outlets and the accessibility of food outlets in a rural community. Shim, Hwang, and Kim [15] found that one of the three household food availability factors was found to significantly differ based on respondent food security status (public food assistance program availability). Further, Shim, Hwang, and Kim [15] find that none of the two household food accessibility factors were found to significantly differ based on respondent food security status. While Shim, Hwang, and Kim [15] did not use a similar measure of the home food environment as was used in this research, it is interesting to note that the authors found little association between food security status and the home food environment. It is also important to reiterate that Shim, Hwang, and Kim [15] sampled a rural population, which makes their research uniquely similar to this study as a study on the association between food security status and the home food environment among a rural sample. As far as it is understood, our research is the only study to directly identify the effect of food security status on a summary measure of total food availability in the home and total food accessibility in the home, as derived from the measures provided by Green and Glanz [4]. Specifically, our study does so through a rural sample of South Carolina residents located in counties with high obesity prevalence.

Our research found that two factors can modify the effect of food security status on the home food environment: food pantry usage and race. This suggests that the effect of food security status on the home food environment differs by respondent food pantry usage (whether the respondent has used a food pantry in the past three months or not). Further, this suggests that the effect of food security status on the home food environment differs by respondent race (whether the respondent identifies as Black or White). Food pantry usage was also found to be a significant effect modifier in the adjusted (final) model. In the final model, the significant interaction term suggests that recent (prior three months) food pantry usage may modify the effect of food security status on the home food environment, especially for food insecure respondents (see stratified analyses in Table S2). Although the extant literature rarely addresses the home food environment within the broader relationship between food security and food access, this finding points to a link. Conceptualizations of the food environment typically focus on the food retail environment [13,36], which largely omits food banks and food pantries. Individuals who are food insecure likely consider food banks and food pantries as part of their food environment, which directly affects their home food environment. Similarly, photovoice research conducted by Heidelberger and Smith [37] found that food pantries are often used to improve food availability in the home food environment among food-insecure households. Taken together, our finding adds evidence that access to food pantries expands the food environment for households facing food insecurity, which may improve their home food environment.

As stated above, Gundersen et al. [11] note that many households below the poverty line are food secure, while many households at or barely above the poverty line are food insecure. The authors reason that this could be due to greater assistance access (in the form of food pantries, SNAP, WIC, etc.) for households below the poverty line as compared with households at or barely above the poverty line. Findings from this study may support the rationale provided by Gundersen et al. [11], as stratified findings shown in Table S2 indicate that among food insecure respondents, not using a food pantry is significantly associated with reduced respondent home food environment scores (95% CI = −2.61–0.56). This suggests that among those food-insecure respondents, actively using a food pantry is associated with improved home food environment scores. This is a meaningful finding because it suggests that local food pantries in the counties under study may be helping to increase household accessibility to healthy foods.

Practical implications from the findings of this research can be found in the fact that food pantry usage influences the association between household food security status and the home food environment. Based on the findings from this study, it can be suggested that policymakers and funders in rural settings may be able to specifically improve the home food environment of food-insecure households with the expansion of food pantry access. Targeting food insecure populations with increased food pantry social assistance may be an efficient way to improve home food environments in rural communities. However, this also requires ensuring food pantries can accommodate food needs that support active and healthy living.

The above practical implications for this research should be reviewed carefully, however. There are opposing sides in the literature as to whether the short-term benefits of food pantry usage outweigh the long-term risks. For example, in several European nations, there is a positive perspective on food pantry usage, particularly because unused food is able to be saved from waste by being provided to low-income households [38]. Further, Garthwaite et al. [39] suggest that food pantry usage in the United Kingdom has been described as a “lifeline” that helps low-income households to maintain food nutrition in the short term. Conversely, Garthwaite et al. [39] note that while food pantries support low-income households in the short-term, food pantries can “unwittingly facilitate the further erosion of income supports to those at the bottom, leading to increased poverty and income inequality and a continued and growing need for charitable food assistance”. Therefore, while the findings of this research suggest that the practical implications for improving the short-term home food environment of rural households would be to increase food pantry access, the literature presents concerns that this may not be a viable long-term solution.

The study results will be valuable for policymakers and funders, specifically those working in rural areas. Learning about the association between food security status and the home food environment can help policymakers and funders to better assess how resources should be distributed in rural communities. Further, this research includes several food access factors so as to identify any effects these variables may have on the relationship between food security status and the home food environment. These additional analyses on food access factors can help stakeholders determine what policies/programs more efficiently address rural household’s home food environments by providing specific examples of what sectors to target (i.e., proximity to a food site, social support from family and/or friends, and public assistance). This research on the association between food security status and the home food environment contributes to the gap in the literature by reviewing the effect(s) that food access factors may exert on this relationship among a rural sample.

As an article that stems from research funded by a program addressing obesity prevention, it is important to note that this article may help to inform the literature on obesity prevention by incorporating factors such as food security status and the home food environment. This is because both food security status [40] and the home food environment [41] have been found to be associated with obesity.

There are many limitations to this research. The first limitation is the relatively small sample size, both in terms of observations and counties. Not only are 436 observations not necessarily enough to accurately identify the food behaviors of rural South Carolinians, but the three rural SC counties under study comprise only three of the twenty-five total rural South Carolina counties [23]. Additional research on rural South Carolina food behaviors would benefit from a larger sample size collected from additional counties. A second limitation is that the home food environment is inconsistently measured throughout the literature. This study incorporates validated measures provided by Green and Glanz [4]; however, it is difficult to compare the findings from this study with home food environment findings from separate studies. Developing a standard measurement for the home food environment, to which this study seeks to contribute, would benefit the food behavior literature and strengthen the research on the home food environment. An additional limitation is the use of zip codes as a random intercept variable. Zip codes cover a relatively large geographical space, which may, in part, explain the low intraclass correlation coefficient (ICC) values. Alternatively, this study would have benefitted from recording specific data collection site locations so that they may be used to account for potential within-site correlations. A fourth limitation of this study is the nature of its cross-sectional design. While using cross-sectional study design methods is beneficial for gathering relatively large sample sizes, it is not possible to draw causal inferences from this research. Another limitation of this study is that the response rate was not obtained during data collection. These data were obtained through convenience sampling, with researchers administering paper copies to participants at on-site locations (such as an office building or a public park). A response rate would have provided the research team with an understanding of potential selection bias, which could be adjusted for in statistical modeling.

## 5. Conclusions and Recommendations

This research fills a gap in the literature on the concept of the home food environment, specifically the effect of food security status on the home food environment among a rural sample. Further, this research identifies an effect that food pantry usage has on the relationship between food security status and the adjusted home food environment. This is a relatively novel concept in the literature, and additional research will be needed to fully identify said effect. Nevertheless, these findings emphasize the value of developing and sustaining food pantries in rural communities in order to improve the short-term home food environment of low-income households. The research provides valuable findings that point to the need to encourage policymakers and funders to consider increasing rural food pantry access.

There is federal support for increasing food pantry access in the United States. For example, the USDA provided about USD 550 million in funding for food pantries and similar programs in 2022 [42]. The goal of this spending is to strengthen the emergency food system (of which food pantries are an integral aspect) in order “to promote food and nutrition security.” [42]. Further, an additional USD 60 million in funding was designated specifically for underserved areas, including rural communities in the United States [42].

This research supports prior literature suggesting that a lower household food security status is associated with a lower home food environment [6,14]. However, findings also suggest that food pantry usage may be used to mitigate said relationship in order to improve the home food environment. Therefore, it is recommended that rural areas with populations experiencing high food insecurity and correspondingly low home food environment scores increase food pantry access locally. The implications of these recommendations emphasize the role that food pantry access can have to improve a household’s short-term home food environment.

## Figures and Tables

**Table 1:** Descriptive statistics.

Frequency Table			
Variables	All Respondents (N = 436)	Food-Secure Respondents (N = 211)	Food-Insecure Respondents (N = 174)
HOME FOOD ENVIRONMENT			
Mean (SD)	10.1 (3.00)	10.6 (2.83)	9.67 (3.11)
Median [min, max]	10.5 [0, 18.0]	10.5 [4.50, 17.5]	9.50 [0, 18.0]
Missing	32 (7.3%)	16 (7.6%)	9 (5.2%)
FOOD SECURITY STATUS			
Food secure	211 (48.4%)	211 (100%)	0 (0%)
High food secure		153 (72.5%)	
Marginal food secure		58 (27.5%)	
Food insecure	174 (39.9%)	0 (0%)	174 (100%)
Low food secure		99 (56.9%)	
Very low food secure		75 (43.1%)	
Missing	51 (11.7%)		
AGE			
Mean (SD)	50.7 (16.7)	51.0 (17.4)	51.8 (15.1)
Median [min, max]	53.0 [17.0, 91.0]	53.0 [18.0, 91.0]	55.5 [20.0, 80.0]
Missing	16 (3.7%)	5 (2.4%)	6 (3.4%)
GENDER			
Female	334 (76.6%)	164 (77.7%)	135 (77.6%)
Male	94 (21.6%)	43 (20.4%)	37 (21.3%)
Missing	8 (1.8%)	4 (1.9%)	2 (1.1%)
ETHNICITY			
Not Hispanic	419 (96.1%)	203 (96.2%)	168 (96.6%)
Hispanic	6 (1.4%)	3 (1.4%)	2 (1.1%)
Missing	11 (2.5%)	5 (2.4%)	4 (2.3%)
RACE			
White	135 (31.0%)	86 (40.8%)	35 (20.1%)
Black	288 (66.1%)	119 (56.4%)	133 (76.4%)
Other	4 (0.9%)	1 (0.5%)	3 (1.7%)
Missing	9 (2.1%)	5 (2.4%)	3 (1.7%)
MARITAL STATUS			
Unmarried	255 (58.5%)	121 (57.3%)	103 (59.2%)
Married	167 (38.3%)	88 (41.7%)	63 (36.2%)
Missing	14 (3.2%)	2 (0.9%)	8 (4.6%)
EMPLOYMENT STATUS			
Employed/self-employed	243 (55.7%)	123 (58.3%)	94 (54.0%)
Other	37 (8.5%)	13 (6.2%)	15 (8.6%)
Unable to work	53 (12.2%)	27 (12.8%)	23 (13.2%)
Retired	80 (18.3%)	40 (19.0%)	31 (17.8%)
Out of work	19 (4.4%)	7 (3.3%)	9 (5.2%)
Missing	4 (0.9%)	1 (0.5%)	2 (1.1%)
INCOME			
Less than 10 k	76 (17.4%)	25 (11.8%)	41 (23.6%)
10–19,999 k	95 (21.8%)	36 (17.1%)	48 (27.6%)
20–34,999 k	103 (23.6%)	40 (19.0%)	53 (30.5%)
35–49,999 k	46 (10.6%)	26 (12.3%)	14 (8.0%)
50–74,999 k	50 (11.5%)	38 (18.0%)	10 (5.7%)
75 k+	47 (10.8%)	38 (18.0%)	3 (1.7%)
Missing	19 (4.4%)	8 (3.8%)	5 (2.9%)
BMI			
Mean (SD)	31.3 (7.29)	31.0 (7.14)	32.1 (7.67)
Median [min, max]	30.8 [17.3, 65.2]	30.8 [17.3, 65.2]	31.1 [17.8, 59.5]
Missing	58 (13.3%)	25 (11.8%)	21 (12.1%)
STORE ACCESS			
Mean (SD)	9.90 (2.09)	9.74 (2.23)	10.2 (1.76)
Median [min, max]	10.0 [4.00, 13.0]	10.0 [4.00, 13.0]	10.0 [6.00, 13.0]
Missing	17 (3.9%)	10 (4.7%)	2 (1.1%)
EDUCATION STATUS			
No HS degree	61 (14.0%)	24 (11.4%)	29 (16.7%)
HS degree through associate degree	250 (57.3%)	115 (54.5%)	105 (60.3%)
Bachelor’s degree	76 (17.4%)	43 (20.4%)	29 (16.7%)
Master’s degree or higher	42 (9.6%)	29 (13.7%)	8 (4.6%)
Missing	7 (1.6%)		3 (1.7%)
NUMBER OF CHILDREN IN THE HOUSEHOLD			
No children	61 (14.0%)	28 (13.3%)	22 (12.6%)
Three or more children	54 (12.4%)	17 (8.1%)	30 (17.2%)
Two children	66 (15.1%)	34 (16.1%)	23 (13.2%)
One child	70 (16.1%)	32 (15.2%)	29 (16.7%)
Missing	185 (42.4%)	100 (47.4%)	70 (40.2%)
FOOD STAMP USAGE			
No household food stamps 12 months	285 (65.4%)	151 (71.6%)	99 (56.9%)
Household food stamps 12 months	137 (31.4%)	52 (24.6%)	72 (41.4%)
Missing	14 (3.2%)	8 (3.8%)	3 (1.7%)
FOOD PANTRY USAGE			
Food pantry usage	192 (44.0%)	66 (31.3%)	108 (62.1%)
No food pantry usage	233 (53.4%)	141 (66.8%)	64 (36.8%)
Missing	11 (2.5%)	4 (1.9%)	2 (1.1%)
FREE MEAL USAGE			
Free meal usage	68 (15.6%)	19 (9.0%)	44 (25.3%)
No free meal usage	355 (81.4%)	187 (88.6%)	124 (71.3%)
Missing	13 (3.0%)	5 (2.4%)	6 (3.4%)
SCHOOL MEAL USAGE			
School meal usage	83 (19.0%)	32 (15.2%)	41 (23.6%)
No school meal usage	337 (77.3%)	173 (82.0%)	127 (73.0%)
Missing	16 (3.7%)	6 (2.8%)	6 (3.4%)
USED HUNTING/FISHING FOR FOOD			
Used hunting/fishing for food	131 (30.0%)	64 (30.3%)	50 (28.7%)
Did not use hunting/fishing for food	297 (68.1%)	144 (68.2%)	121 (69.5%)
Missing	8 (1.8%)	3 (1.4%)	3 (1.7%)
USED FRIENDS/NEIGHBORS/COWORKERS FOR FOOD			
Used friends/neighbors/coworkers for food	127 (29.1%)	42 (19.9%)	73 (42.0%)
Did not use friends/neighbors/coworkers for food	296 (67.9%)	165 (78.2%)	96 (55.2%)
Missing	13 (3.0%)	4 (1.9%)	5 (2.9%)
USED RELATIVES OUTSIDE THE HOME FOR FOOD			
Used relatives outside the home for food	161 (36.9%)	61 (28.9%)	84 (48.3%)
Did not use relatives outside the home for food	261 (59.9%)	145 (68.7%)	84 (48.3%)
Missing	14 (3.2%)	5 (2.4%)	6 (3.4%)
NUMBER OF SNAP VENDORS			
Mean (SD)	13.4 (7.11)	12.9 (7.29)	14.1 (6.94)
Median [min, max]	15.0 [1.00, 21.0]	15.0 [1.00, 21.0]	15.0 [1.00, 21.0]
Missing	33 (7.6%)	14 (6.6%)	16 (9.2%)
HEALTHY SNAP VENDORS			
Mean (SD)	1.96 (1.32)	1.89 (1.34)	2.06 (1.32)
Median [min, max]	2.00 [0, 4.00]	2.00 [0, 4.00]	2.00 [0, 4.00]
Missing	33 (7.6%)	14 (6.6%)	16 (9.2%)
UNHEALTHY SNAP VENDORS			
Mean (SD)	11.5 (6.15)	11.0 (6.35)	12.0 (5.96)
Median [min, max]	13.0 [0, 19.0]	13.0 [1.00, 19.0]	13.0 [0, 19.0]
Missing	33 (7.6%)	14 (6.6%)	16 (9.2%)

Note: SD = standard deviation.

**Table 2:** Effect modifier selection.

Covariate × Food Security Status	Interaction Term *p*-Value	Effect Modifier
Age	0.37	No
BMI	0.47	No
Children In The Household	0.52	No
Education	0.64	No
Employment	0.99	No
Ethnicity	0.47	No
Gender	0.86	No
Income	0.3	No
Marriage	0.94	No
Race	0.003	Yes
Food Pantry Usage	0.0008	Yes
Free Meal Usage	0.14	No
School Lunch Usage	0.7	No
Hunting/Fishing Usage	0.61	No
Friends, Coworkers, Neighbors Usage	0.13	No
Relatives Outside The Home Usage	0.74	No
Food Stamp Usage	0.9	No
Store Access	0.6	No
Number Of SNAP Vendors In The Zip Code	0.84	No
Number Of Healthy SNAP Vendors In The Zip Code	0.61	No
Number Of Unhealthy SNAP Vendors In The Zip Code	0.9	No

**Table 3:** Fully unadjusted models with home food environment as the outcome variable (all respondents).

Variables	Estimate (CI)	*p*-Value
FOOD SECURITY STATUS		
Food secure	-	-
Food insecure	−0.74 (−1.38–−0.1)	0.023 *
AGE	0.02 (0.01–0.04)	0.011 *
GENDER		
Female	-	-
Male	−0.34 (−1.09–0.4)	0.366
ETHNICITY		
Not Hispanic	-	-
Hispanic	−0.25 (−2.67–2.18)	0.840
RACE		
White	-	-
Black	0.07 (−0.64–0.80)	0.827
MARITAL STATUS		
Not married	-	-
Married	0.59 (−0.02–1.21)	0.608
EMPLOYMENT STATUS		
Employed/self-employed	-	-
Other	0.56 (−0.53–1.65)	0.313
Unable to work	−0.46 (−1.42–0.51)	0.354
Retired	0.08 (−0.74–0.89)	0.855
Out of work	0.83 (−0.62–2.29)	0.261
INCOME		
Less than 10 k	-	-
10–19,999 k	0.36 (−0.60–1.32)	0.459
20–34,999 k	0.08 (−0.86–1.01)	0.871
35–49,999 k	1.23 (0.05–2.42)	0.041 *
50–74,999 k	1.15 (0.01–2.30)	0.048 *
75 k+	2.41 (1.24–3.57)	0.00005 ***
BMI	0 (−0.04–0.04)	0.987
EDUCATION STATUS		
No HS degree	-	-
HS/vocational/associate/professional degree	0.34 (−0.52–1.20)	0.440
Bachelor’s degree	1.87 (0.85–2.9)	0.0004 ***
Master’s degree or higher	1.7 (0.48–2.91)	0.006 **
NUMBER OF CHILDREN IN THE HOUSEHOLD		
No children	-	-
One child	−0.59 (−1.72–0.54)	0.302
Two children	−0.25 (−1.38–0.89)	0.667
Three or more children	−0.71 (−1.92–0.5)	0.248
FOOD PANTRY USAGE		
Food pantry usage	-	-
No food pantry usage	0.06 (−0.57–0.69)	0.846
FREE MEAL USAGE		
Free meal usage	-	-
No free meal usage	−0.01 (−0.83–0.82)	0.987
SCHOOL MEAL USAGE		
School meal usage	-	-
No school meal usage	0.49 (−0.29–1.27)	0.213
USED HUNTING/FISHING FOR FOOD		
Used hunting/fishing for food	-	-
Did not use hunting/fishing for food	−0.16 (−0.81–0.5)	0.635
USED FRIENDS/NEIGHBORS/COWORKERS FOR FOOD		
Used friends/neighbors/coworkers for food	-	-
Did not use friends/neighbors/coworkers for food	0.15 (−0.51–0.82)	0.646
USED RELATIVES FOR FOOD		
Used relatives outside the home	-	-
Did not use relatives outside the home for food	0.15 (−0.48–0.78)	0.632
NUMBER OF SNAP VENDORS	−0.03 (−0.15–0.1)	0.588
HEALTHY SNAP VENDORS	−0.19 (−0.88–0.51)	0.495
UNHEALTHY SNAP VENDORS	−0.03 (−0.18–0.12)	0.631
STORE ACCESS	0.02 (−0.13–0.17)	0.798
FOOD STAMP USAGE		
No household food stamps 12 months	-	-
Household food stamps 12 months	−1.07 (−1.72–−0.41)	0.001 ***

Note: CI = confidence interval; *** *p* < 0.005, ** *p* < 0.01, * *p* < 0.05.

**Table 4:** Fully adjusted model with home food environment as the outcome variable (all respondents).

Variables	Estimate (CI)	*p*-Value
FOOD SECURITY STATUS		
Food secure	-	-
Food insecure	−2.05 (−2.79–2.38)	0.875
AGE	0.02 (−0.03–0.07)	0.389
GENDER		
Female	-	-
Male	−0.35 (−1.97–1.25)	0.661
ETHNICITY		
Not Hispanic	-	-
Hispanic	−0.92 (−7.45–5.79)	0.803
RACE		
White	-	-
Black	0.58 (−7.55–5.70)	0.782
MARITAL STATUS		
Not married	-	-
Married	−0.20 (−1.62–1.22)	0.779
EMPLOYMENT STATUS		
Employed/self-employed	-	-
Other	1.22 (−1.23–3.67)	0.325
Unable to work	0.40 (−1.76–2.57)	0.711
Retired	1.02 (−0.89–3.10)	0.277
Out of work	−0.42 (−3.45–2.60)	0.782
INCOME		
Less than 10 k	-	-
10–19,999 k	0.88 (−1.10–2.86)	0.380
20–34,999 k	−0.50 (−2.67–1.66)	0.646
35–49,999 k	0.61 (−1.97–3.20)	0.638
50–74,999 k	0.548 (−2.28–3.38)	0.701
75 k+	2.80 (−0.17–5.78)	0.065
BMI	−0.01 (−0.08–0.06)	0.753
STORE ACCESS	−0.26 (−0.56–0.034)	0.082
EDUCATION STATUS		
No HS degree	-	-
HS/vocational/associate/professional degree	1.37 (−0.42–3.17)	0.132
Bachelor’s degree	2.80 (0.51–5.09)	0.016 *
Master’s degree or higher	3.34 (0.70–5.97)	0.013 *
NUMBER OF CHILDREN IN THE HOUSEHOLD		
Zero children	-	-
Three or more children	0.99 (−1.04–3.03)	0.337
Two children	0.24 (−1.47–1.96)	0.778
One child	0.11 (−1.54–1.78)	0.888
FOOD STAMP USAGE		
No household food stamps 12 months	-	-
Household food stamps 12 months	−0.08 (−1.45–1.28)	0.903
FOOD PANTRY USAGE		
Food pantry usage	-	-
No food pantry usage	−1.09 (−0.67–2.86)	0.224
FREE MEAL USAGE		
Free meal usage	-	-
No free meal usage	0.16 (−1.52–1.85)	0.846
SCHOOL MEAL USAGE		
School meal usage	-	-
No school meal usage	0.003 (−1.51–1.51)	0.996
USED HUNTING/FISHING FOR FOOD		
Used hunting/fishing for food	-	-
Did not use hunting/fishing for food	−0.35 (−1.66–0.95)	0.593
USED FRIENDS/NEIGHBORS/COWORKERS FOR FOOD		
Used friends/neighbors/coworkers for food	-	-
Did not use friends/neighbors/coworkers for food	−0.02 (−1.79–1.75)	0.980
USED RELATIVES OUTSIDE THE HOME FOR FOOD		
Used relatives outisde the home for food	-	-
Did not use relatives outside the home for food	−0.18 (−1.78–1.41)	0.822
NUMBER OF SNAP VENDORS	0.01 (−0.12–0.15)	0.831
HEALTHY SNAP VENDORS	−0.25 (−0.94–0.42)	0.455
FOOD SECURITY STATUS AND RACE INTERACTION TERM	1.70 (−0.89–4.30)	0.196
FOOD SECURITY STATUS AND FOOD PANTRY USAGE INTERACTION TERM	−2.58 (−4.96–−0.19)	0.034 *

Note: CI = confidence interval; * *p* < 0.05.

## Data Availability

Due to privacy and ethical concerns, neither the data nor the source of the data can be made available.

## Supplementary Materials

The following supporting information can be downloaded at: https://www.mdpi.com/article/10.3390/nu15183918/s1, Supplementary Materials S1 (Table S1): Fully Unadjusted Models with Home Food Environment as the Outcome Variable (Food Secure Respondents); Supplementary Materials S2 (Table S2): Fully Unadjusted Models with Home Food Environment as the Outcome Variable (Food Insecure Respondents); Supplementary Materials S3 (Table S3): Fully Adjusted Model with Home Food Environment as the Outcome Variable (Food Secure Respondents); Supplementary Materials S4 (Table S4): Fully Adjusted Model with Home Food Environment as the Outcome Variable (Food Insecure Respondents); Supplementary Materials S5 (Table S5): Intraclass Correlation Coefficients (ICC’s) (All Respondents); Supplementary Materials S6 (R-Code); Supplementary Materials S7 (Food Access and Health Assessment—HOP Counties).
