# Supplementary material for: The Association between Food Security Status and the Home Food Environment among a Sample of Rural South Carolina Residents
Source: Nutrients. 2023 Sep 9;15(18):3918. doi: 10.3390/nu15183918 (PMC10534860; doi:10.3390/nu15183918)
Supplement: Supplementary file 1 [file nutrients-15-03918-s001.zip › nutrients-2533546-supplementary.pdf]

Supplementary Materials

Supplemntary Materials S1

Table S1. Fully Unadjusted Models with Home Food Environment as the Outcome Variable (Food Secure Respondents).

VARIABLES	ESTIMATE (CI)	P-VALUE
FOOD SECURITY STATUS		
High food secure	-	-
Marginal food secure	-0.92 (-1.83 - 0)	0.051
AGE	0.01 (-0.02 - 0.03)	0.434
GENDER		
Female	-	-
Male	-0.33 (-1.38 - 0.73)	0.544
ETHNICITY		
Not Hispanic	-	-
Hispanic	-0.93 (-4.2 - 2.34)	0.575
RACE		
White	-	-
Black	-0.82 (-1.74 - 0.09)	0.079
MARITAL STATUS		
Married	-	-
Unmarried	0.59 (-0.25 - 1.44)	0.166
EMPLOYMENT STATUS		
Other	-	-
Employed/self-employed	-0.70 (-2.41 – 1.00)	0.415
Unable to work	-1.34 (-3.37 – 0.68)	0.192
Retired	-0.94 (-2.82 – 0.93)	0.321
Out of work	0.27 (-2.39 – 2.94)	0.839
INCOME		
75k+	-	-
Less than 10k	-1.77 (-3.26 – -0.29)	0.019*
10-19,999k	-2.20 (-3.55 – -0.86)	0.001**
20-34,999k	-2.47 (-3.78 – -1.16)	0.0002***
35-49,999k	-1.67 (-3.23 – -0.11)	0.035*
50-74,999k	-1.24 (-2.55 – 0.06)	0.063
BMI	0.006 (-0.05 – 0.06)	0.844
EDUCATION STATUS		
No HS degree	-	-
HS/vocational/associate/professional degree	-0.37 (-1.69 – 0.94)	0.573
Bachelor’s degree	1.03 (-0.43 – 2.51)	0.166
Master’s degree or higher	1.08 (-0.53 – 2.71)	0.187
NUMBER OF CHILDREN IN THE HOUSEHOLD		
No children	-	-
One child	-0.39 (-2.49 – 1.30)	0.626
Two children	0.52 (-1.08 – 2.12)	0.522
Three or more children	-0.59 (-2.49 – 1.30)	0.535
FOOD PANTRY USAGE		
Food pantry usage	-	-

**No food pantry usage**	-0.67 (-1.56 – 0.21)	0.135
**FREE MEAL USAGE**		
**Free meal usage**	-	-
**No free meal usage**	-0.80 (-2.27 – 0.66)	0.281
**SCHOOL MEAL USAGE**		
**School meal usage**	-	-
**No school meal usage**	-0.24 (-1.42 – 0.94)	0.690
**USED HUNTING/FISHING FOR FOOD**		
**Used hunting/fishing for food**	-	-
**Did not use hunting/fishing for food**	0.34 (-0.55 – 1.24)	0.449
**USED FRIENDS/NEIGHBORS/COWORKERS FOR FOOD**		
**Used friends/neighbors/coworkers for food**	-	-
**Did not use friends/neighbors/coworkers for food**	-0.50 (-1.55 0.54)	0.344
**USED RELATIVES FOR FOOD**		
**Used relatives outside the home**	-	-
**Did not use relatives outside the home for food**	0.04 (-0.88 – 0.96)	0.928
**NUMBER OF SNAP VENDORS**	-0.01 (-0.14 – 0.10)	0.729
**HEALTHY SNAP VENDORS**	-0.10 (-0.75 – 0.55)	0.700
**UNHEALTHY SNAP VENDORS**	-0.01 (-0.16 – 0.13)	0.752
**STORE ACCESS**	0.006 (-0.18 – 0.19)	0.947
**FOOD STAMP USAGE**		
**No household food stamps 12 months**	-	-
**Household food stamps 12 months**	-0.91 (-1.89 – 0.06)	0.066

Note: CI = confidence interval; *** $p < 0.005$, ** $p < 0.01$, * $p < 0.05$.

Supplementaey Materials S2

Table S2. Fully Unadjusted Models with Home Food Environment as the Outcome Variable (Food Insecure Respondents).

VARIABLES	ESTIMATE (CI)	P-VALUE
FOOD SECURITY STATUS		
Low food secure	-	-
Very low food secure	0.7 (-0.31 - 1.72)	0.17
AGE	0.02 (-0.01 - 0.06)	0.176
GENDER		
Female	-	-
Male	-0.38 (-1.63 - 0.87)	0.549
ETHNICITY		
Not Hispanic	-	-
Hispanic	1.03 (-3.48 - 5.54)	0.651
RACE		
White	-	-
Black	1.46 (0.21 – 2.72)	0.021
MARITAL STATUS		
Not married	-	-
Married	0.64 (-0.41 - 1.68)	0.230
EMPLOYMENT STATUS		
Employed/self-employed	-	-
Other	0.77 (-1.11 – 2.66)	0.419
Unable to work	-0.44 (-1.99 – -1.12)	0.582
Retired	-0.19 (-1.60 – 1.21)	0.784
Out of work	0.50 (-1.73 – 2.74)	0.655
INCOME		
Less than 10k	-	-
10-19,999k	1.52 (0.06 - 2.98)	0.037*
20-34,999k	0.59 (-0.8 - 1.98)	0.394
35-49,999k	2.44 (0.38 - 4.49)	0.018*
50-74,999k	2.04 (-0.28 - 4.36)	0.084*
75k+	0.46 (-4.16 - 5.07)	0.841
BMI	0.04 (-0.03 - 0.11)	0.258
EDUCATION STATUS		
No HS degree	-	-
HS/vocational/associate/professional degree	0.7 (-0.68 - 2.09)	0.318
Bachelor's degree	2.21 (0.5 - 3.92)	0.017*
Master's degree or higher	1.63 (-0.89 - 4.15)	0.202
NUMBER OF CHILDREN IN THE HOUSEHOLD		
No children	-	-
One child	-0.08 (-2.02 - 1.85)	0.932
Two children	-0.4 (-2.42 - 1.61)	0.691
Three or more children	0.52 (-1.42 - 2.45)	0.596
FOOD PANTRY USAGE		
Food pantry usage	-	-
No food pantry usage	-1.59 (-2.61 – -0.56)	0.002***
FREE MEAL USAGE		
Free meal usage		

**No free meal usage**	-0.66 (-1.83 - 0.51)	0.265
**SCHOOL MEAL USAGE**		
**School meal usage**		
**No school meal usage**	-0.24 (-1.45 - 0.97)	0.694
**USED HUNTING/FISHING FOR FOOD**		
**Used hunting/fishing for food**	-	-
**Did not use hunting/fishing for food**	-0.02 (-1.13 - 1.1)	0.977
**USED FRIENDS/NEIGHBORS/COWORKERS FOR FOOD**		
**Used friends/neighbors/coworkers for food**	-	-
**Did not use friends/neighbors/coworkers for food**	-0.71 (-1.75 - 0.32)	0.175
**USED RELATIVES FOR FOOD**		
**Used relatives outside the home**	-	-
**Did not use relatives outside the home for food**	-0.29 (-1.32 - 0.75)	0.585
**NUMBER OF SNAP VENDORS**	-0.05 (-0.12 - 0.02)	0.178
**HEALTHY SNAP VENDORS**	-0.3 (-0.68 - 0.08)	0.117
**UNHEALTHY SNAP VENDORS**	-0.05 (-0.15 - 0.05)	0.253
**STORE ACCESS**	0.07 (-0.21 - 0.36)	0.608
**FOOD STAMP USAGE**		
**No household food stamps 12 months**	-	-
**Household food stamps 12 months**	-0.86 (-1.9 - 0.18)	0.102

Note: CI = confidence interval; *** $p < 0.005$, * $p < 0.05$.

Supplementary Materials S3

Table S3. Fully Adjusted Model with Home Food Environment as the Outcome Variable (Food Secure Respondents).

VARIABLES	ESTIMATE (CI)	P-VALUE
FOOD SECURITY STATUS		
High food secure	-	-
Marginal food secure	0.20 (-2.0 – 2.49)	0.858
AGE	0.01 (-0.08 - 0.1)	0.810
GENDER		
Female	-	-
Male	0.01 (-3.04 - 3.05)	0.996
ETHNICITY		
Not Hispanic	-	-
Hispanic	-1.03 (-9.31 - 7.26)	0.803
RACE		
White	-	-
Black	0.55 (-2.42 - 3.52)	0.709
MARITAL STATUS		
Not married	-	-
Married	0.44 (-2.21 - 3.1)	0.737
EMPLOYMENT STATUS		
Employed/self-employed	-	-
Other	3.36 (-1.5 - 8.22)	0.169
Unable to work	1.91 (-2.54 - 6.36)	0.391
Retired	2.44 (-1.27 - 6.15)	0.192
Out of work	0.69 (-8.26 - 9.63)	0.877
INCOME		
Less than 10k	-	-
10-19,999k	1.29 (-3.83 - 6.41)	0.613
20-34,999k	-0.04 (-5.03 - 4.95)	0.987
35-49,999k	0.86 (-5.52 - 7.24)	0.787
50-74,999k	1.06 (-5.15 - 7.27)	0.732
75k+	3.25 (-3.01 - 9.5)	0.300
BMI	0 (-0.12 - 0.12)	0.995
STORE ACCESS	-0.12 (-0.61 - 0.37)	0.619
EDUCATION STATUS		
No HS degree	-	-
HS/vocational/associate/professional degree	0.07 (-3.38 - 3.52)	0.968
Bachelor's degree	1.23 (-2.91 - 5.36)	0.552
Master's degree or higher	2.52 (-2.01 - 7.06)	0.267
NUMBER OF CHILDREN IN THE HOUSEHOLD		
Zero children	-	-
One child	-0.29 (-3.08 - 2.49)	0.831
Two children	0.07 (-3.06 - 3.19)	0.965
Three or more children	0.21 (-3.66 - 4.08)	0.912
FOOD STAMP USAGE		
No household food stamps 12 months	-	-
Household food stamps 12 months	-0.05 (-2.59 - 2.49)	0.971
FOOD PANTRY USAGE		

**Food pantry usage**	-	-
**No food pantry usage**	1.42 (-1.1 - 3.95)	0.262
**FREE MEAL USAGE**		
**Free meal usage**	-	-
**No free meal usage**	0.1 (-3.66 - 3.87)	0.955
**SCHOOL MEAL USAGE**		
**School meal usage**	-	-
**No school meal usage**	-0.95 (-3.7 - 1.81)	0.492
**USED HUNTING/FISHING FOR FOOD**		
**Used hunting/fishing for food**	-	-
**Did not use hunting/fishing for food**	-0.57 (-2.75 - 1.61)	0.599
**USED FRIENDS/NEIGHBORS/COWORKERS FOR FOOD**		
**Used friends/neighbors/coworkers for food**	-	-
**Did not use friends/neighbors/coworkers for food**	0.95 (-1.97 - 3.88)	0.514
**USED RELATIVES OUTSIDE THE HOME FOR FOOD**		
**Used relatives outisde the home for food**	-	-
**Did not use relatives outside the home for food**	-0.4 (-2.94 - 2.13)	0.749
**NUMBER OF SNAP VENDORS**	-0.03 (-0.29 - 0.24)	0.831
**HEALTHY SNAP VENDORS**	0.02 (-1.21 - 1.25)	0.975

Note: CI = confidence interval.

Supplementary Materials S4

Table S4. Fully Adjusted Model with Home Food Environment as the Outcome Variable (Food Insecure Respondents).

VARIABLES	ESTIMATE (CI)	P-VALUE
FOOD SECURITY STATUS		
Low food secure	-	-
Very low food secure	0.51 (-1.78 - 2.79)	0.656
AGE	0.07 (-0.04 - 0.17)	0.194
GENDER		
Female	-	-
Male	-0.05 (-2.64 - 2.54)	0.970
ETHNICITY		
Not Hispanic	-	-
Hispanic	1.89 (-1.06 - 4.84)	0.201
RACE		
White	-	-
Black	-0.94 (-3.39 - 1.51)	0.443
MARITAL STATUS		
Not married	-	-
Married	-0.79 (-4.73 - 3.16)	0.69
EMPLOYMENT STATUS		
Employed/self-employed	-	-
Other	-0.06 (-3.89 - 3.77)	0.974
Unable to work	-0.71 (-4.09 - 2.67)	0.672
Retired	-2.29 (-6.18 - 1.59)	0.239
Out of work	-0.78 (-4.72 – 3.15)	0.688
INCOME		
Less than 10k	-	-
10-19,999k	0.91 (-2.07 – 3.90)	0.539
20-34,999k	-1.29 (-4.63 - 2.04)	0.438
35-49,999k	1.24 (-2.88 - 5.37)	0.545
50-74,999k	1.27 (-3.79 - 6.32)	0.614
BMI	-0.02 (-0.16 - 0.12)	0.774
STORE ACCESS	-0.47 (-1.1 - 0.15)	0.131
EDUCATION STATUS		
No HS degree	-	-
HS/vocational/associate/professional degree	3.31 (0.38 - 6.24)	0.027*
Bachelor's degree	5.87 (1.65 - 10.08)	0.007**
Master's degree or higher	4.46 (-0.22 - 9.13)	0.061
NUMBER OF CHILDREN IN THE HOUSEHOLD		
Zero children	-	-
One child	1.64 (-1.69 - 4.97)	0.325
Two children	1.24 (-2.04 – 4.53)	0.449
Three or more children	2.06 (-1.62 - 5.73)	0.264
FOOD STAMP USAGE		
No household food stamps 12 months	-	-
Household food stamps 12 months	0.14 (-1.98 - 2.26)	0.894
FOOD PANTRY USAGE		
Food pantry usage	-	-

**No food pantry usage**	-2.18 (-4.85 - 0.48)	0.105
**FREE MEAL USAGE**		
**Free meal usage**		
**No free meal usage**	0.62 (-2.41 - 3.64)	0.682
**SCHOOL MEAL USAGE**		
**School meal usage**		
**No school meal usage**	0.99 (-1.88 - 3.86)	0.487
**USED HUNTING/FISHING FOR FOOD**		
**Used hunting/fishing for food**		
**Did not use hunting/fishing for food**	-0.66 (-3.06 - 1.74)	0.582
**USED FRIENDS/NEIGHBORS/COWORKERS FOR FOOD**		
**Used friends/neighbors/coworkers for food**		
**Did not use friends/neighbors/coworkers for food**	-1.89 (-5.34 - 1.56)	0.273
**USED RELATIVES OUTSIDE THE HOME FOR FOOD**		
**Used relatives outisde the home for food**		
**Did not use relatives outside the home for food**	0.74 (-2.55 - 4.03)	0.651
**NUMBER OF SNAP VENDORS**	0.15 (-0.14 - 0.44)	0.293
**HEALTHY SNAP VENDORS**	-0.99 (-2.29 - 0.31)	0.130

Note: CI = confidence interval; ** $p < 0.01$, * $p < 0.05$.

Supplementary Materials S5

Table S5. Intraclass Correlation Coefficients (ICC's) (All Respondents).

MEASURE	ICC VALUE	95% CI
**Food Security Status**	0.062	(0.003 – 0.149)
**Age**	0.106	(0.014 – 0.219)
**Gender**	0.082	(0.005 – 0.184)
**Ethnicity**	0.080	(0.007 – 0.184)
**Race**	0.093	(0.013 – 0.212)
**Marital Status**	0.068	(0.005 – 0.177)
**Employment Status**	0.084	(0.009 – 0.193)
**Income**	0.033	(0.003 – 0.113)
**BMI**	0.056	(0.004 – 0.147)
**Store Access**	0.087	(0.016 – 0.246)
**Education Status**	0.068	(0.008 – 0.171)
**Number Of Children In The Household**	0.075	(0.005 – 0.213)
**Household Food Stamps 12 Months**	0.083	(0.008 – 0.171)
**Food Pantry Usage**	0.086	(0.010 – 0.186)
**Free Meal Usage**	0.094	(0.013 – 0.209)
**School Meal Usage**	0.078	(0.009 – 0.191)
**Used Hunting/Fishing For Food**	0.084	(0.007 – 0.208)
**Used Friends/Neighbors/Coworkers For Food**	0.087	(0.009 – 0.176)
**Used Relatives Outside The Home For Food**	0.078	(0.013 – 0.192)
**Number Of SNAP Vendors**	0.086	(0.012 – 0.206)
**Healthy SNAP Vendors**	0.085	(0.007 – 0.223)
**Unhealthy SNAP Vendors**	0.088	(0.007 – 0.168)

Note: ICC = intraclass correlation coefficient; CI = confidence interval.

Note: Table S5 shows the intraclass correlation coefficients (ICC's) for each of the unadjusted LMM models. The minimum ICC value found is 0.033 and the maximum ICC value found is 0.106. The median ICC value found is 0.0835. The measures found in Table S5 are the individual predictors that were included in each unadjusted model. For example, the corresponding ICC for the unadjusted model which identified the association between food security status and the home food environment (with zip code as the random intercept variable) is 0.062. This suggests that for this unadjusted model, about 6.2% of the variation in home food environment scores can be attributed to participant zip code.

Supplementary Materials S6

R-Code

```
library("haven")
library("cat")
library("car")
library("table1")
library("tidyverse")
library("tibble")
library("gapminder")
library("highcharter")
library("knitr")
library("kableExtra")
library("dplyr")
library("epiDisplay")
library("expss")
library ("nlme")
library ("tidyr")
library ("dplyr")
library ("ggplot2")
library ("readxl")
library ("emmeans")
library ("sjstats")
library ("lme4")
library ("lmerTest")
library ("MuMIn")
library ("xtable")
library ("broom")
library ("broom.mixed")
library ("readxl")
library ("janitor")
library ("irr")
library ("performance")
library ("boot")
library ("psych")

#Import Data
HOP_SurveyData <- read_sav("~/HOP/Survey Data/HOP_SurveyData.sav")
View(HOP_SurveyData)
View(HOP_SurveyData_SNAP_Vendors)

#Merge Data
#SNAP Vendors
SNAP_Vendors <- read_excel("C:\\Users\\hossf\\OneDrive - Clemson University\\Documents\\HOP\\Michelle Eichinger\\SNAP Vendors\\SNAP_Store_Locations_Comparison\\SNAP_Vendors_by_ZipCode.xlsx")
colnames(HOP_SurveyData)
colnames(SNAP_Vendors)
HOP_SurveyData_SNAP_Vendors <- merge(HOP_SurveyData, SNAP_Vendors, by="ZipCode", all.x=TRUE)
colnames(HOP_SurveyData_SNAP_Vendors)
```

```
HOP_Study_SNAP_Vendors <- filter(SNAP_Vendors,ZipCode%in%HOP_SurveyData_SNAP_Vendors$ZipCode)
View(SNAP_Vendors)
View(HOP_SurveyData_SNAP_Vendors)
View(HOP_Study_SNAP_Vendors)

#Descriptive Statistics and Frequencies
table1(~Home_Food_Environment+factor(sum_foodsecurity_binary)+Q53_1_A+factor(Male)+factor(Hispanic)+factor(RACE)+factor(Marriage)+factor(EmploymentStatus)+factor(Income)+bmi_continuous+sum_storeaccess+factor(EducationStatus1)+factor(ChildrenHousehold)+factor(FFS)+factor(Q6_1_1)+factor(Q6_2_2)+factor(Q6_3_3)+factor(Q6_4_4)+factor(Q6_5_5)+factor(Q6_6_6)+factor(ZipCode)+Number_of_SNAP_Vendors+Healthy_SNAP_Vendors+Unhealthy_SNAP_Vendors,data=HOP_SurveyData_SNAP_Vendors, caption=caption)
table1(~Number_of_SNAP_Vendors,data=SNAP_Vendors)
table1(~HOP_Study_SNAP_Vendors$Number_of_SNAP_Vendors)
table1(~HOP_Study_SNAP_Vendors$Healthy_SNAP_Vendors)
table1(~HOP_Study_SNAP_Vendors$Unhealthy_SNAP_Vendors)

#Frequencies for Relevant Variables
HOP_SurveyData_SNAP_Vendors$sum_foodsecurity_binary <- factor(HOP_SurveyData_SNAP_Vendors$sum_foodsecurity_binary, levels=c(1,0), labels=c("Food Secure", "Food Insecure"))
HOP_SurveyData_SNAP_Vendors$Male <- factor(HOP_SurveyData_SNAP_Vendors$Male, levels=c(1,0), labels=c("Male","Female"))
HOP_SurveyData_SNAP_Vendors$Hispanic <- factor(HOP_SurveyData_SNAP_Vendors$Hispanic, levels=c(1,0), labels=c("Hispanic","Not Hispanic"))
HOP_SurveyData_SNAP_Vendors$RACE <- factor(HOP_SurveyData_SNAP_Vendors$RACE, levels=c(2,1), labels=c("Black","White"))
HOP_SurveyData_SNAP_Vendors$Marriage <- factor(HOP_SurveyData_SNAP_Vendors$Marriage, levels=c(1,0), labels=c("Married","Unmarried"))
HOP_SurveyData_SNAP_Vendors$EmploymentStatus <- factor(HOP_SurveyData_SNAP_Vendors$EmploymentStatus, levels=c(10,4,3,2,1), labels=c("Other","Unable to Work","Retired","Out of Work","Employed/Self-Employed"))
HOP_SurveyData_SNAP_Vendors$Income <- factor(HOP_SurveyData_SNAP_Vendors$Income, levels=c(6,5,4,3,2,1), labels=c("75K+","50-74,999K","35-49,999K","20-34,999K","10-19,999K","Less than 10K"))
HOP_SurveyData_SNAP_Vendors$EducationStatus1 <- factor(HOP_SurveyData_SNAP_Vendors$EducationStatus1, levels=c(4,3,2,1), labels=c("Master's Degree or Higher","Bachelor's Degree","HS Degree through Associate Degree","No HS Degree"))
HOP_SurveyData_SNAP_Vendors$ChildrenHousehold <- factor(HOP_SurveyData_SNAP_Vendors$ChildrenHousehold, levels=c(3,2,1,0), labels=c("Three or More Children","Two Children","One Child","No Children"))
HOP_SurveyData_SNAP_Vendors$FFS <- factor(HOP_SurveyData_SNAP_Vendors$FFS, levels=c(1,0), labels=c("Household Food Stamps 12 Months","No Household Food Stamps 12 Months"))
HOP_SurveyData_SNAP_Vendors$Q6_1_1 <- factor(HOP_SurveyData_SNAP_Vendors$Q6_1_1, levels=c(1,0), labels=c("Food Pantry Usage","No Food Pantry Usage"))
HOP_SurveyData_SNAP_Vendors$Q6_2_2 <- factor(HOP_SurveyData_SNAP_Vendors$Q6_2_2, levels=c(1,0), labels=c("Free Meal Usage","No Free Meal Usage"))
HOP_SurveyData_SNAP_Vendors$Q6_3_3 <- factor(HOP_SurveyData_SNAP_Vendors$Q6_3_3, levels=c(1,0), labels=c("School Meal Usage","No School Meal Usage"))
HOP_SurveyData_SNAP_Vendors$Q6_4_4 <- factor(HOP_SurveyData_SNAP_Vendors$Q6_4_4, levels=c(1,0), labels=c("Hunting/Fishing","No Hunting/Fishing"))
HOP_SurveyData_SNAP_Vendors$Q6_5_5 <- factor(HOP_SurveyData_SNAP_Vendors$Q6_5_5, levels=c(1,0), labels=c("Used Friends/Neighbors for Food","Did not use Friends/Neighbors for Food"))
```

```
HOP_SurveyData_SNAP_Vendors$Q6_6_6 <- factor(HOP_SurveyData_SNAP_Vendors$Q6_6_6, levels=c(1,0), labels=c("Used Relatives for Food","Did not use Relatives for Food"))
caption <- "Frequency Table"

#Regression Modeling
#Reference Setting
HOP_SurveyData_SNAP_Vendors$sum_foodsecurity_binary <- as.factor(HOP_SurveyData_SNAP_Vendors$sum_foodsecurity_binary)
levels(HOP_SurveyData_SNAP_Vendors$sum_foodsecurity_binary)
HOP_SurveyData_SNAP_Vendors$sum_foodsecurity_binary <- relevel(HOP_SurveyData_SNAP_Vendors$sum_foodsecurity_binary, ref = "Food Secure")
class(HOP_SurveyData_SNAP_Vendors$sum_foodsecurity_binary)
table(HOP_SurveyData_SNAP_Vendors$sum_foodsecurity_binary)

HOP_SurveyData_SNAP_Vendors$Male <- as.factor(HOP_SurveyData_SNAP_Vendors$Male)
levels(HOP_SurveyData_SNAP_Vendors$Male)
HOP_SurveyData_SNAP_Vendors$Male <- relevel(HOP_SurveyData_SNAP_Vendors$Male, ref = "Female")

HOP_SurveyData_SNAP_Vendors$Hispanic <- as.factor(HOP_SurveyData_SNAP_Vendors$Hispanic)
levels(HOP_SurveyData_SNAP_Vendors$Hispanic)
HOP_SurveyData_SNAP_Vendors$Hispanic <- relevel(HOP_SurveyData_SNAP_Vendors$Hispanic, ref = "Not Hispanic")

HOP_SurveyData_SNAP_Vendors$RACE <- as.factor(HOP_SurveyData_SNAP_Vendors$RACE)
levels(HOP_SurveyData_SNAP_Vendors$RACE)
HOP_SurveyData_SNAP_Vendors$RACE <- relevel(HOP_SurveyData_SNAP_Vendors$RACE, ref = "White")

HOP_SurveyData_SNAP_Vendors$Marriage <- as.factor(HOP_SurveyData_SNAP_Vendors$Marriage)
levels(HOP_SurveyData_SNAP_Vendors$Marriage)
HOP_SurveyData_SNAP_Vendors$Marriage <- relevel(HOP_SurveyData_SNAP_Vendors$Marriage, ref = "Unmarried")
class(HOP_SurveyData_SNAP_Vendors$Marriage)
table(HOP_SurveyData_SNAP_Vendors$Marriage)

HOP_SurveyData_SNAP_Vendors$EmploymentStatus <- as.factor(HOP_SurveyData_SNAP_Vendors$EmploymentStatus)
levels(HOP_SurveyData_SNAP_Vendors$EmploymentStatus)
HOP_SurveyData_SNAP_Vendors$EmploymentStatus <- relevel(HOP_SurveyData_SNAP_Vendors$EmploymentStatus, ref = "Employed/Self-Employed")
class(HOP_SurveyData_SNAP_Vendors$EmploymentStatus)
table(HOP_SurveyData_SNAP_Vendors$EmploymentStatus)

HOP_SurveyData_SNAP_Vendors$Income <- factor(HOP_SurveyData_SNAP_Vendors$Income,levels = c("Less than 10K","10-19,999K","20-34,999K","35-49,999K","50-74,999K","75K+"))
levels(HOP_SurveyData_SNAP_Vendors$Income)
HOP_SurveyData_SNAP_Vendors$Income <- relevel(HOP_SurveyData_SNAP_Vendors$Income, ref = "Less than 10K")
class(HOP_SurveyData_SNAP_Vendors$Income)
table(HOP_SurveyData_SNAP_Vendors$Income)

HOP_SurveyData_SNAP_Vendors$EducationStatus1 <- as.factor(HOP_SurveyData_SNAP_Vendors$EducationStatus1)
levels(HOP_SurveyData_SNAP_Vendors$EducationStatus1)
```

```
HOP_SurveyData_SNAP_Vendors$EducationStatus1 <- relevel(HOP_SurveyData_SNAP_Vendors$EducationStatus1, ref = "No HS Degree")
class(HOP_SurveyData_SNAP_Vendors$EducationStatus1)
table(HOP_SurveyData_SNAP_Vendors$EducationStatus1)

HOP_SurveyData_SNAP_Vendors$ChildrenHousehold <- as.factor(HOP_SurveyData_SNAP_Vendors$ChildrenHousehold)
levels(HOP_SurveyData_SNAP_Vendors$ChildrenHousehold)
HOP_SurveyData_SNAP_Vendors$ChildrenHousehold <- relevel(HOP_SurveyData_SNAP_Vendors$ChildrenHousehold, ref = "No Children")
class(HOP_SurveyData_SNAP_Vendors$ChildrenHousehold)
table(HOP_SurveyData_SNAP_Vendors$ChildrenHousehold)

HOP_SurveyData_SNAP_Vendors$FFS <- as.factor(HOP_SurveyData_SNAP_Vendors$FFS)
levels(HOP_SurveyData_SNAP_Vendors$FFS)
HOP_SurveyData_SNAP_Vendors$FFS <- relevel(HOP_SurveyData_SNAP_Vendors$FFS, ref = "No Household Food Stamps 12 Months")
class(HOP_SurveyData_SNAP_Vendors$FFS)
table(HOP_SurveyData_SNAP_Vendors$FFS)

#Interaction Terms included in Modeling
InteractionAge <- lmer(Home_Food_Environment ~ sum_foodsecurity_binary + Q53_1_A + sum_foodsecurity_binary*Q53_1_A + (1|ZipCode), data=HOP_SurveyData_SNAP_Vendors)
InteractionGender <- lmer(Home_Food_Environment ~ sum_foodsecurity_binary + Male + sum_foodsecurity_binary*Male + (1|ZipCode), data=HOP_SurveyData_SNAP_Vendors)
InteractionEthnicity <- lmer(Home_Food_Environment ~ sum_foodsecurity_binary + Hispanic + sum_foodsecurity_binary*Hispanic + (1|ZipCode), data=HOP_SurveyData_SNAP_Vendors)
InteractionRACE <- lmer(Home_Food_Environment ~ sum_foodsecurity_binary + RACE + sum_foodsecurity_binary*RACE + (1|ZipCode), data=HOP_SurveyData_SNAP_Vendors)
InteractionMarriage <- lmer(Home_Food_Environment ~ sum_foodsecurity_binary + Marriage + sum_foodsecurity_binary*Marriage + (1|ZipCode), data=HOP_SurveyData_SNAP_Vendors)
InteractionEmployment <- lmer(Home_Food_Environment ~ sum_foodsecurity_binary + EmploymentStatus + sum_foodsecurity_binary*EmploymentStatus + (1|ZipCode), data=HOP_SurveyData_SNAP_Vendors)
InteractionIncome <- lmer(Home_Food_Environment ~ sum_foodsecurity_binary + Income + sum_foodsecurity_binary*Income + (1|ZipCode), data=HOP_SurveyData_SNAP_Vendors)
InteractionBMI <- lmer(Home_Food_Environment ~ sum_foodsecurity_binary + bmi_continuous + sum_foodsecurity_binary*bmi_continuous + (1|ZipCode), data=HOP_SurveyData_SNAP_Vendors)
InteractionStoreAccess <- lmer(Home_Food_Environment ~ sum_foodsecurity_binary + sum_storeaccess + sum_foodsecurity_binary*sum_storeaccess + (1|ZipCode), data=HOP_SurveyData_SNAP_Vendors)
InteractionEducation <- lmer(Home_Food_Environment ~ sum_foodsecurity_binary + EducationStatus1 + sum_foodsecurity_binary*EducationStatus1 + (1|ZipCode), data=HOP_SurveyData_SNAP_Vendors)
InteractionChildren <- lmer(Home_Food_Environment ~ sum_foodsecurity_binary + ChildrenHousehold + sum_foodsecurity_binary*ChildrenHousehold + (1|ZipCode), data=HOP_SurveyData_SNAP_Vendors)
InteractionFoodStamps <- lmer(Home_Food_Environment ~ sum_foodsecurity_binary + FFS + sum_foodsecurity_binary*FFS + (1|ZipCode), data=HOP_SurveyData_SNAP_Vendors)
InteractionFoodPantry <- lmer(Home_Food_Environment ~ sum_foodsecurity_binary + Q6_1_1 + sum_foodsecurity_binary*Q6_1_1 + (1|ZipCode), data=HOP_SurveyData_SNAP_Vendors)
InteractionFreeMeal <- lmer(Home_Food_Environment ~ sum_foodsecurity_binary + Q6_2_2 + sum_foodsecurity_binary*Q6_2_2 + (1|ZipCode), data=HOP_SurveyData_SNAP_Vendors)
```

```
InteractionSchoolMeal <- lmer(Home_Food_Environment ~ sum_foodsecurity_binary + Q6_3_3 + sum_foodsecurity_binary*Q6_3_3 + (1|ZipCode), data=HOP_SurveyData_SNAP_Vendors)
InteractionHuntFish <- lmer(Home_Food_Environment ~ sum_foodsecurity_binary + Q6_4_4 + sum_foodsecurity_binary*Q6_4_4 + (1|ZipCode), data=HOP_SurveyData_SNAP_Vendors)
InteractionFriendsNeighbors <- lmer(Home_Food_Environment ~ sum_foodsecurity_binary + Q6_5_5 + sum_foodsecurity_binary*Q6_5_5 + (1|ZipCode), data=HOP_SurveyData_SNAP_Vendors)
InteractionRelatives <- lmer(Home_Food_Environment ~ sum_foodsecurity_binary + Q6_6_6 + sum_foodsecurity_binary*Q6_6_6 + (1|ZipCode), data=HOP_SurveyData_SNAP_Vendors)
InteractionNumberofSNAPVendors <- lmer(Home_Food_Environment ~ sum_foodsecurity_binary + Number_of_SNAP_Vendors + sum_foodsecurity_binary*Number_of_SNAP_Vendors + (1|ZipCode), data=HOP_SurveyData_SNAP_Vendors)
InteractionHealthySNAP <- lmer(Home_Food_Environment ~ sum_foodsecurity_binary + Healthy_SNAP_Vendors + sum_foodsecurity_binary*Healthy_SNAP_Vendors + (1|ZipCode), data=HOP_SurveyData_SNAP_Vendors)
InteractionUnhealthySNAP <- lmer(Home_Food_Environment ~ sum_foodsecurity_binary + Unhealthy_SNAP_Vendors + sum_foodsecurity_binary*Unhealthy_SNAP_Vendors + (1|ZipCode), data=HOP_SurveyData_SNAP_Vendors)

anova(InteractionAge)
anova(InteractionGender)
anova(InteractionEthnicity)
anova(InteractionRACE)
anova(InteractionMarriage)
anova(InteractionEmployment)
anova(InteractionIncome)
anova(InteractionBMI)
anova(InteractionStoreAccess)
anova(InteractionEducation)
anova(InteractionChildren)
anova(InteractionFoodStamps)
anova(InteractionFoodPantry)
anova(InteractionFreeMeal)
anova(InteractionSchoolMeal)
anova(InteractionHuntFish)
anova(InteractionFriendsNeighbors)
anova(InteractionRelatives)
anova(InteractionNumberofSNAPVendors)
anova(InteractionHealthySNAP)
anova(InteractionUnhealthySNAP)

#Analysis
model100 <- lmer(Home_Food_Environment ~ sum_foodsecurity_binary + (1|ZipCode), data = HOP_SurveyData_SNAP_Vendors)
anova(model100)
is_singular <- isSingular(model100)
print(is_singular)
summary(model100)
print(model100)
vif(model100)
icc(model100)
```

```
model1 <- lmer(Home_Food_Environment ~ sum_foodsecurity_binary + Q53_1_A + Male + Hispanic + RACE + Marriage + EmploymentStatus + Income + bmi_continuous + sum_storeaccess + EducationStatus1 + ChildrenHousehold + FFS + Q6_1_1 + Q6_2_2 + Q6_3_3 + Q6_4_4 + Q6_5_5 + Q6_6_6 + Number_of_SNAP_Vendors + Healthy_SNAP_Vendors + Unhealthy_SNAP_Vendors + sum_foodsecurity_binary*RACE + sum_foodsecurity_binary*Q6_1_1 + (1|ZipCode), data = HOP_SurveyData_SNAP_Vendors)
anova(model1)
is_singular <- isSingular(model1)
print(is_singular)
summary(model1)
print(model1)
vif(model1)
icc(model1)

CI.A1 = as.matrix(tidy(model1 ,conf.int=TRUE,exponentiate=FALSE,effects="fixed") %>% dplyr::select(term, estimate, conf.low, conf.high, p.value))

FullyAdjustedModel = rbind(CI.A1[-1, ])
FullyAdjustedModel = data.frame(FullyAdjustedModel)
print(FullyAdjustedModel)
write.csv(FullyAdjustedModel,"C:\\Users\\User\\OneDrive - Clemson University\\Documents\\Courses\\PhD\\Summer 2023\\Independent Study\\FullyAdjustedModel.csv")

model0 <- lmer(Home_Food_Environment ~ sum_foodsecurity_binary + (1|ZipCode), data = HOP_SurveyData_SNAP_Vendors)
anova(model0)
summary(model0)
confint(model0)
print(model0)
icc(model0, ci = 0.95)

CI.Z = as.matrix(tidy(model0 ,conf.int=TRUE,exponentiate=FALSE,effects="fixed") %>% dplyr::select(term, estimate, conf.low, conf.high, p.value))

model5 <- lmer(Home_Food_Environment ~ Q53_1_A + (1|ZipCode), data = HOP_SurveyData_SNAP_Vendors)
anova(model5)
summary(model5)
confint(model5)
print(model5)
icc(model5, ci = 0.95)

CI.A = as.matrix(tidy(model5 ,conf.int=TRUE,exponentiate=FALSE,effects="fixed") %>% dplyr::select(term, estimate, conf.low, conf.high, p.value))

model7 <- lmer(Home_Food_Environment ~ Male + (1|ZipCode), data = HOP_SurveyData_SNAP_Vendors)
anova(model7)
summary(model7)
confint(model7)
print(model7)
icc(model7, ci = 0.95)
```

```
CI.B = as.matrix(tidy(model7 ,conf.int=TRUE,exponentiate=FALSE,effects="fixed") %>% dplyr::select(term, estimate, conf.low, conf.high, p.value))

model9 <- lmer(Home_Food_Environment ~ Hispanic + (1|ZipCode), data = HOP_SurveyData_SNAP_Vendors)
anova(model9)
summary(model9)
confint(model9)
print(model9)
icc(model9, ci = 0.95)

CI.C = as.matrix(tidy(model9 ,conf.int=TRUE,exponentiate=FALSE,effects="fixed") %>% dplyr::select(term, estimate, conf.low, conf.high, p.value))

model11 <- lmer(Home_Food_Environment ~ RACE + (1|ZipCode), data = HOP_SurveyData_SNAP_Vendors)
anova(model11)
summary(model11)
confint(model11)
print(model11)
icc(model11, ci = 0.95)

CI.D = as.matrix(tidy(model11 ,conf.int=TRUE,exponentiate=FALSE,effects="fixed") %>% dplyr::select(term, estimate, conf.low, conf.high, p.value))

model13 <- lmer(Home_Food_Environment ~ Marriage + (1|ZipCode), data = HOP_SurveyData_SNAP_Vendors)
anova(model13)
summary(model13)
confint(model13)
print(model13)
icc(model13, ci = 0.95)

CI.1 = as.matrix(tidy(model13 ,conf.int=TRUE,exponentiate=FALSE,effects="fixed") %>% dplyr::select(term, estimate, conf.low, conf.high, p.value))

model15 <- lmer(Home_Food_Environment ~ EmploymentStatus + (1|ZipCode), data = HOP_SurveyData_SNAP_Vendors)
anova(model15)
summary(model15)
confint(model15)
print(model15)
icc(model15, ci = 0.95)

HOP_SurveyData_SNAP_Vendors$EmploymentStatus <-
as.factor(HOP_SurveyData_SNAP_Vendors$EmploymentStatus)

CI.2 = as.matrix(tidy(model15 ,conf.int=TRUE,exponentiate=FALSE,effects="fixed") %>% dplyr::select(term, estimate, conf.low, conf.high, p.value))

model17 <- lmer(Home_Food_Environment ~ Income + (1|ZipCode), data = HOP_SurveyData_SNAP_Vendors)
anova(model17)
```

```
summary(model17)
confint(model17)
print(model17)
icc(model17, ci = 0.95)

HOP_SurveyData_SNAP_Vendors$Income <- as.factor(HOP_SurveyData_SNAP_Vendors$Income)

CI.3 = as.matrix(tidy(model17 ,conf.int=TRUE,exponentiate=FALSE,effects="fixed") %>% dplyr::select(term, estimate, conf.low, conf.high, p.value))
print(CI.3)

model19 <- lmer(Home_Food_Environment ~ bmi_continuous + (1|ZipCode), data = HOP_SurveyData_SNAP_Vendors)
anova(model19)
summary(model19)
confint(model19)
print(model19)
icc(model19, ci = 0.95)

CI.4 = as.matrix(tidy(model19 ,conf.int=TRUE,exponentiate=FALSE,effects="fixed") %>% dplyr::select(term, estimate, conf.low, conf.high, p.value))

model21 <- lmer(Home_Food_Environment ~ EducationStatus1 + (1|ZipCode), data = HOP_SurveyData_SNAP_Vendors)
anova(model21)
summary(model21)
confint(model21)
print(model21)
icc(model21, ci = 0.95)

HOP_SurveyData_SNAP_Vendors$EducationStatus1 <- as.factor(HOP_SurveyData_SNAP_Vendors$EducationStatus1)

CI.5 = as.matrix(tidy(model21 ,conf.int=TRUE,exponentiate=FALSE,effects="fixed") %>% dplyr::select(term, estimate, conf.low, conf.high, p.value))

model23 <- lmer(Home_Food_Environment ~ ChildrenHousehold + (1|ZipCode), data = HOP_SurveyData_SNAP_Vendors)
anova(model23)
summary(model23)
confint(model23)
print(model23)
icc(model23, ci = 0.95)

HOP_SurveyData_SNAP_Vendors$ChildrenHousehold <- as.factor(HOP_SurveyData_SNAP_Vendors$ChildrenHousehold)

CI.6 = as.matrix(tidy(model23 ,conf.int=TRUE,exponentiate=FALSE,effects="fixed") %>% dplyr::select(term, estimate, conf.low, conf.high, p.value))

model25 <- lmer(Home_Food_Environment ~ Q6_1_1 + (1|ZipCode), data = HOP_SurveyData_SNAP_Vendors)
anova(model25)
```

```
summary(model25)
confint(model25)
print(model25)
icc(model25, ci = 0.95)

CI.7 = as.matrix(tidy(model25 ,conf.int=TRUE,exponentiate=FALSE,effects="fixed") %>% dplyr::select(term, estimate, conf.low, conf.high, p.value))

model27 <- lmer(Home_Food_Environment ~ Q6_2_2 + (1|ZipCode), data = HOP_SurveyData_SNAP_Vendors)
anova(model27)
summary(model27)
confint(model27)
print(model27)
icc(model27, ci = 0.95)

CI.8 = as.matrix(tidy(model27 ,conf.int=TRUE,exponentiate=FALSE,effects="fixed") %>% dplyr::select(term, estimate, conf.low, conf.high, p.value))

model29 <- lmer(Home_Food_Environment ~ Q6_3_3 + (1|ZipCode), data = HOP_SurveyData_SNAP_Vendors)
anova(model29)
summary(model29)
confint(model29)
print(model29)
icc(model29, ci = 0.95)

CI.9 = as.matrix(tidy(model29 ,conf.int=TRUE,exponentiate=FALSE,effects="fixed") %>% dplyr::select(term, estimate, conf.low, conf.high, p.value))

model31 <- lmer(Home_Food_Environment ~ Q6_4_4 + (1|ZipCode), data = HOP_SurveyData_SNAP_Vendors)
anova(model31)
summary(model31)
confint(model31)
print(model31)
icc(model31, ci = 0.95)

CI.10 = as.matrix(tidy(model31 ,conf.int=TRUE,exponentiate=FALSE,effects="fixed") %>% dplyr::select(term, estimate, conf.low, conf.high, p.value))

model33 <- lmer(Home_Food_Environment ~ Q6_5_5 + (1|ZipCode), data = HOP_SurveyData_SNAP_Vendors)
anova(model33)
summary(model33)
confint(model33)
print(model33)
icc(model33, ci = 0.95)

CI.H = as.matrix(tidy(model33 ,conf.int=TRUE,exponentiate=FALSE,effects="fixed") %>% dplyr::select(term, estimate, conf.low, conf.high, p.value))

model35 <- lmer(Home_Food_Environment ~ Q6_6_6 + (1|ZipCode), data = HOP_SurveyData_SNAP_Vendors)
```

```
anova(model35)
summary(model35)
confint(model35)
print(model35)
icc(model35, ci = 0.95)

CI.11 = as.matrix(tidy(model35 ,conf.int=TRUE,exponentiate=FALSE,effects="fixed") %>% dplyr::select(term, estimate, conf.low, conf.high, p.value))

model37 <- lmer(Home_Food_Environment ~ Number_of_SNAP_Vendors + (1|ZipCode), data = HOP_SurveyData_SNAP_Vendors)
anova(model37)
summary(model37)
confint(model37)
print(model37)
icc(model37, ci = 0.95)

CI.12 = as.matrix(tidy(model37 ,conf.int=TRUE,exponentiate=FALSE,effects="fixed") %>% dplyr::select(term, estimate, conf.low, conf.high, p.value))

model39 <- lmer(Home_Food_Environment ~ Healthy_SNAP_Vendors + (1|ZipCode), data = HOP_SurveyData_SNAP_Vendors)
anova(model39)
summary(model39)
confint(model39)
print(model39)
icc(model39, ci = 0.95)

CI.13 = as.matrix(tidy(model39 ,conf.int=TRUE,exponentiate=FALSE,effects="fixed") %>% dplyr::select(term, estimate, conf.low, conf.high, p.value))

model41 <- lmer(Home_Food_Environment ~ Unhealthy_SNAP_Vendors + (1|ZipCode), data = HOP_SurveyData_SNAP_Vendors)
anova(model41)
summary(model41)
confint(model41)
icc(model41, ci = 0.95)

CI.14 = as.matrix(tidy(model41 ,conf.int=TRUE,exponentiate=FALSE,effects="fixed") %>% dplyr::select(term, estimate, conf.low, conf.high, p.value))

model43 <- lmer(Home_Food_Environment ~ sum_storeaccess + (1|ZipCode), data = HOP_SurveyData_SNAP_Vendors)
anova(model43)
summary(model43)
confint(model43)
print(model43)
icc(model43, ci = 0.95)
```

```
CI.15 = as.matrix(tidy(model43 ,conf.int=TRUE,exponentiate=FALSE,effects="fixed") %>% dplyr::select(term, estimate, conf.low, conf.high, p.value))

model45 <- lmer(Home_Food_Environment ~ FFS + (1|ZipCode), data = HOP_SurveyData_SNAP_Vendors)
anova(model45)
summary(model45)
confint(model45)
print(model45)
icc(model45, ci = 0.95)

CI.16 = as.matrix(tidy(model45 ,conf.int=TRUE,exponentiate=FALSE,effects="fixed") %>% dplyr::select(term, estimate, conf.low, conf.high, p.value))

FullyUnadjustedModel = rbind(CI.Z[-1, ], CI.A[-1, ], CI.B[-1, ], CI.C[-1, ], CI.D[-1, ], CI.1[-1, ], CI.2[-1, ], CI.3[-1, ], CI.4[-1, ],CI.5[-1, ],CI.6[-1, ],CI.7[-1, ],CI.8[-1, ],CI.9[-1, ],CI.10[-1, ],CI.H[-1, ],CI.11[-1, ],CI.12[-1, ],CI.13[-1, ],CI.14[-1, ],CI.15[-1, ],CI.16[-1, ])
FullyUnadjustedModel = data.frame(FullyUnadjustedModel)
print(FullyUnadjustedModel)
write.csv(FullyUnadjustedModel,"C:\\Users\\User\\OneDrive - Clemson University\\Documents\\Courses\\PhD\\Summer 2023\\Independent Study\\FullyUnadjustedModel.csv")
```

Supplementary Materials S7

FOOD ACCESS AND HEALTH ASSESSMENT - HOP Counties

County Codes: 5-Hampton, 7-Lee, 8-Marion

County Code (ex. 1) ______
Last 4 digits of interviewee phone number (ex. 4564) ______
Interview Initials (ex. SG): ______

Screening Section

1. In what county do you currently live?
   - ○ Hampton
   - ○ Lee
   - ○ Marion
2. What is your zip code? ____________ 3. Where do you shop for food?
   a. City/Town: ____________
   b. Stores: ____________
   c. Of these, which store do you shop/buy most of your food? ____________

Food Access

4. Please indicate how often you or members of your household shopped for food during the last 3 months at the following different types of food stores.

	I never bought food here	I sometimes bought food here	I often bought my food here	I always bought my food here	Do not know	Refuse to answer
Supermarket or grocery store (Aldi, Ingles, BILO, Publix, Walmart, etc.)	○	○	○	○	○	○
Partial market (Walgreens, Dollar Store, CVS, etc.)	○	○	○	○	○	○
Specialty store (Asian food market, Mexican food market)	○	○	○	○	○	○
Convenience store, carryout, corner store (gas station)	○	○	○	○	○	○
Fast food restaurant, food truck (counter service)	○	○	○	○	○	○

					○	○
Farmers' market or produce stand	○	○	○	○	○	○
Big box stores (Costco, Sam's Club, etc.)	○	○	○	○	○	○

5. Over the last 3 months, how often did you use the following types of transportation to go food shopping?

	Never	Sometimes	Most of the time	All of the time	Don't know	Refuse to answer
My own car	○	○	○	○	○	○
Getting a ride with someone I know	○	○	○	○	○	○
Public transportation	○	○	○	○	○	○
Taxi service, Uber, or Lyft	○	○	○	○	○	○
Bike	○	○	○	○	○	○
Walking	○	○	○	○	○	○
Senior bus	○	○	○	○	○	○
Other	○	○	○	○	○	○

6. In the past 3 months, how often has your household depended on any of the following food sources?

	Never	Less than once a month	Once a month	2-3 times a month	Every week	Don't know	Refuse to answer

Food pantry
Free meal (Salvation Army, Community Center)
Federal school lunch or breakfast program
Hunting or fishing
Friends, co-workers, neighbors
Relatives outside of the home
Community or personal garden
Senior Center Food Distribution programs
Other

7. In the past 12 months, did you/any family members living here receive food stamp benefits/ [SNAP or food stamp benefits]?

- ○ Yes
- ○ No
- ○ Don't Know

8. Did you/any family members living here receive food stamp benefits/ [SNAP or food stamp benefits] in the last 30 days?

- ○ Yes
- ○ No
- ○ Don't Know

9. Thinking about the store where you buy most of your food, how do you usually travel to this store?

- ○ Car
- ○ Other form of transportation

10. About how long would it take you to get from your home to the store where you buy most of your food, if you walked there?

- ○ 10 minutes or less
- ○ 11-20 minutes
- ○ 21-30 minutes
- ○ More than 30 minutes

11. How important are each of the following factors in your decision to shop at the store where you buy most of your food?

	Not at all important	A little important	Somewhat important	Very important
Near your home	○	○	○	○
Near or on the way to other places where you spend time	○	○	○	○

Food Security

12. Please state how easy it is to access (purchase or get) the following items in your local community.

	Not easy	Somewhat easy	Very easy	Do not know	Refuse to answer
Fresh fruits and vegetables	○	○	○	○	○
Locally grown or home-made food items	○	○	○	○	○
Food support services (food pantry, free meals)	○	○	○	○	○
Farmer's market or produce stands	○	○	○	○	○
Affordable food	○	○	○	○	○
Healthy food	○	○	○	○	○

Fast food	○	○	○	○	○

13. Do you have access to food through (check all that apply): Your own garden

- ☐ A neighbor's garden
- ☐ A community garden
- ☐

14. In the past 3 months, have you or anyone in your household had to choose between buying the food you need or paying for any of the following? Check all that apply.

- ☐ Medicine or medicinal care
- ☐ Utilities (electricity or cell phone) Rent or mortgage
- ☐ Gas or fuel for vehicle
- ☐ Other bills (childcare) Do not know
- ☐ **Refuse to answer N/A**
- ☐
- ☐
- ☐

Now I'm going to read you several statements that people have made about their food situation. For these statements, please tell me whether the statement was often true, sometimes true, or never true for (you/your household) in the last 3 months.

15. The first statement is, "The food that (I/we) bought just didn't last, and (I/we) didn't have money to get more." Was that often, sometimes, or never true for (you/your household) in the last 3 months?

- ○ Often true
- ○ Sometimes true
- ○ Never True
- ○ **Don't know Refuse to answer**
- ○

16. "(I/we) couldn't afford to eat balanced meals." Was that often, sometimes, or never true for (you/your household) in the last 3 months? Often true

- ○ Sometimes true
- ○ Never True
- ○ **Don't know Refuse to answer**
- ○

17. In the last 3 months, did (you/you or other adults in your household) ever cut the size of your meals or skip meals because there wasn't enough money for food? Yes No

- ○
- ○ **Don't know**
- ○

Go to question 18 if they respond yes to question 17. Otherwise, skip to question 19.

18. How often did this happen - almost every month, some months but not every month, or in only 1 or 2 months?

- ○ Almost every month
- ○ Some months but not every month Only 1 or 2 months
- ○ **Don't know**
- ○

19. In the last 3 months, did you ever eat less than you felt you should because there wasn't enough money for food?

○ Yes No

○ Don't know

○ **20.** In the last 3 months, were you ever hungry but didn't eat because there wasn't enough money for food? Yes No

○ Don't know

○

○

Eating Behaviors

21. Now think about the foods you ate or drank during the past month, that is, the past 30 days, including meals and snacks. (Only enter a response in one column per item)

	Times per day	Times per week	Times per month
Not including juices, how often did you eat fruit? You can tell me times per day, times per week or times per month.			
Not including fruit-flavored drinks or fruit juices with added sugar, how often did you drink 100% fruit juice such as apple or orange juice?			
How often did you eat a green leafy or lettuce salad, with or without other vegetables?			
How often did you eat any kind of fried potatoes, including French fries, home fries, or hash browns?			
How often did you eat any other kind of potatoes, or sweet potatoes, such as baked, boiled, mashed potatoes, or potato salad?			
Not including lettuce salads and potatoes, how often did you eat other vegetables?			

22. Please indicate whether each of these food items were available in your home in the past week:

(a-j)	Yes	No
Bananas		
Apples		
Grapes		
Carrots		
Tomatoes		
Dark leafy greens (spinach, collards, kale, etc.)		

Reduced-fat hot dogs		
Whole-grain bread		
Low-fat milk		
Diet soda		

23. Please indicate whether each of these food items were available in your home in the past week:

(a-f)	Yes	No
Candy or cookies		
Snack chips (potato chips, corn chips, tortilla chips, etc.)		
Regular whole milk		
Regular (non-diet) soda		
Regular hot dogs		
White bread		

24. In your home, how often do you…

a. Have fruits and vegetables in the refrigerator?

○ Never or rarely ○ Sometimes
○ Often
○ Almost always

b. Have fruit available in a bowl or on the counter?

○ Never or rarely ○ Sometimes
○ Often
○ Almost always

25. In your home, how often do you…

a. Have candy or chips available to eat?

○ Never or rarely ○ Sometimes
○ Often
○ Almost always

b. Have ice-cream, cake, pastries, or ready-to-eat sweet baked goods (cookies, brownies, etc.)?

Never or rarely Sometimes
Often
Almost always

BRFSS Physical Activity

26. During the past month, other than your regular job, did you participate in any physical activities or exercise such as running, calisthenics, golf, gardening or walking exercise?

Yes No

Don't know Refused

Go to question 27 if they respond yes to question 26. Otherwise, skip to question 30.

27. What type of physical activity or exercise did you spend the most time doing during the last month? (select the type that best fits what they tell you)

03 Backpacking 05 Basketball 07 Bicycling 08 Boating (Canoeing, rowing, kayaking, sailing for pleasure or camping) 16 Fishing from river bank or boat 17 Frisbee 18 Gardening (spading, weeding, digging, filling) 19 Golf (with motorized cart) 20 Golf (without motorized cart) 22 Hiking – cross-country

24 Horseback riding 25 Hunting large game – deer, elk 26 Hunting small game – quail 28 Jogging 29 Lacrosse 31 Mowing lawn 36 Raking lawn/trimming

hedges 37 Running 43 Skateboarding 51 Soccer 52 Softball/Baseball 55 Stream fishing in waders 57 Swimming 61 Tennis 64 Walking 67 Weight lifting 69 Yoga

○ 71 Childcare ○ 72

Farm/Ranch Work
(caring for livestock, stacking hay, etc.)

○ 74 Karate/Martial Arts ○ 76

Yard work
(cutting/gathering wood, trimming,

etc.) ○ 77 Other/Not listed ○

77 Refused

28. How many times per week or per month did you take part in this activity?

Times per week __________

Times per month __________

○ Don’t know ○ Refused

29. And when you took part in this activity, for how many minutes or hours did you usually keep at it? Hours __________

Minutes __________

○ Don’t know ○ Refused

Physical Activity

30. In the past 7 days, did you walk for transportation [This is walking you might have done to travel to and from work, to do errands, or to go from place to place. Include walking to or from a bus, train, or bus stop]?

○ Yes

○ No

○ Don’t know

○ Refused

Go to question 31 if they respond yes to question 30. Otherwise, skip to question 30.

31. In the past 7 days, how many days did you walk for transportation?

○ 0 days

○ 1 day

○ 2 days

○ 3 days

○ 4 days

○ 5 days

○ 6 days

○ 7 days

○ Don’t know

○ Refused

32. On average, how many times per day did you walk for transportation? Times per day __________

33. How long did that walk take? On average, how long did those walks take?

Hours __________

Minutes __________

34. Sometimes you may walk for fun, relaxation, exercise, or to walk the dog. In the past 7 days, did you walk for any of these reasons? Yes No

Don't know Refused

Go to question 35 if they respond yes to question 34. Otherwise, skip to question 38.

35. In the past 7 days, how many days did you walk for leisure or exercise? 0 days

1 day

2 days

3 days

4 days

5 days

6 days

7 days

Don't know Refused

36. On average, how many times per day did you walk for leisure or exercise? Times per day __________

37. How long did that walk take? On average, how long did those walks take? Hours _________

Minutes __________

Neighborhood

The next questions are about where you live. By where you live we mean in your neighborhood or near your home.

38. Where you live, are there roads, sidewalks, paths or trails where you can walk? Yes No

Don't know Refused

39. Are there shops, stores, or markets that you can walk to?

Yes No

Don’t know Refused

40. Are there bus or transit stops that you can walk to? Yes No

Don’t know Refused

41. Are there places like movies, libraries, or places of worship that you can walk to?

Yes No

Don’t know Refused

42. Are there places that you can walk to that help you relax, clear your mind, and reduce stress?

Yes

No

Don’t know Refused

43. Where you live, do most streets have sidewalks? Yes No

Don’t know

Refused

44. Does traffic make it unsafe for you to walk?

Yes No

Don’t know Refused

45. Does crime make it unsafe for you to walk?

Yes

No

Don't know Refused

46. Do dogs or other animals make it unsafe for you to walk? Yes

No

Don't know Refused

47. How often are there people walking within sight of your home? Would you say every day, every 2 to 3 days, about once a week, or less than once a week? Every day

Every 2 to 3 days

About once a week

Less than once a week

Don't know Refused

Stress

48. Thinking about the amount of stress in your life, would you say that most days are: Not at all stressful

Not very stressful

A bit stressful

Quite a bit stressful

Very Stressful

49. In the last month, how often have you….

	Never	Almost never	Sometimes	Fairly often	Very Often
been upset because of something that happened unexpectedly?	○	○	○	○	○

felt that you were unable to control the important things in your life?	○	○	○	○	○
felt nervous and “stressed”?	○	○	○	○	○
felt confident about your ability to handle your personal problems?	○	○	○	○	○
felt that things were going your way?	○	○	○	○	○
found that you could not cope with all the things that you had to do?	○	○	○	○	○
been able to control irritations in your life?	○	○	○	○	○
felt that you were on top of things?	○	○	○	○	○
been angered because of things that were outside of your control?	○	○	○	○	○
felt difficulties were piling up so high that you could not overcome them?	○	○	○	○	○

Community Belonging

50. How would you describe your sense of belonging to your local community? Would you say it is:

- ○ Very strong
- ○ Somewhat strong
- ○ Somewhat weak
- ○ Very weak

51. How strongly would you agree or disagree that:

	Strongly disagree	Disagree	Agree	Strongly Agree
People around here are willing to help their neighbors	○	○	○	○
This is a close-knit neighborhood	○	○	○	○
People in this neighborhood can be trusted	○	○	○	○
People in this neighborhood generally don't get along with each other	○	○	○	○
People in this neighborhood do not share the same values	○	○	○	○

52. How likely would you say it is that neighbors know each other well enough that they could be counted on to intervene in various ways if:

	Very Unlikely	Unlikely	Likely	Very Likely
Children were skipping school and hanging out on a street corner	○	○	○	○
Children were spray-painting graffiti on a local building	○	○	○	○
Children were showing disrespect to an adult	○	○	○	○
A fight broke out in front of their house	○	○	○	○

The fire station or other community resource, such as park or community center, closest to their home was being threatened with budget cuts	○	○	○	○

Demographics

I will ask you some questions about yourself in the next section. We include these questions so that we can compare health indicators by groups.

53. What is your age (in years)?

○ Years __________

○ **Don't know Refused**

54. What is your sex?

○ Male

○ Female

○

○ **Neither of these Refused**

55. Are you of Hispanic, Latino/a, or Spanish origin?

○

○

○

○

Yes

No

Don't know Refused

56. Which one of these groups would you say best represents your race? (Check all that apply)

White

Black or African American

American Indian or Alaska Native

Asian

Pacific Islander

Don't know Refused

57. Are you,

○ Married
○ Widowed
○ Divorced
○ Never married
○ A member of an unmarried couple
○ Refused

58. Are you currently,

○ Employed for wages
○ Self-employed
○ Out of work for 1 year or less
○ Out of work for more than 1 year
○ A homemaker
○ A student
○ Retired
○ Unable to work
○ **Refused**

59. Is your household income from all sources,

○ Less than $10,000
○ $10,000 to less than $15,000
○ $15,000 to less than $20,000
○ $20,000 to less than $25,000
○ $25,000 to less than $35,000
○ $35,000 to less than $50,000
○ $50,000 to less than $75,000
○ $75,000 or more

60. About how much do you weigh without shoes? Pounds __________

○ **Don't know**
○ **Refused**

61. About how tall are you without shoes?

Feet __________

Inches __________

○ **Don't know**
○ **Refused**

62. What is the number of people in your household?

Adults __________

Children (below 18 years) __________

63. What is the highest level of school you have completed or the highest degree you have received? ○ Never attended/kindergarten only

○ Grade 1-11 ○ 12th grade, no diploma ○ GED or equivalent ○ High school graduate ○ Some college, no degree

○ Associate degree: occupational, technical, or vocational program ○ Associate degree: academic program ○ Bachelor's Degree (ex. BA, AB, BS, BBA) ○ Master's Degree (MA, MS, MEng, MEd, MBA) ○ Professional School (MD, DDS, DVM, JD) ○ Doctoral Degree (ex. PhD, EdD)

○ Don't know

○ Refused

64. How did you hear about this survey?
